# Age-specific case data reveal varying dengue transmission intensity in US states and territories

**DOI:** 10.1371/journal.pntd.0011143

**Published:** 2024-03-01

**Authors:** Sarah Kada, Gabriela Paz-Bailey, Laura E. Adams, Michael A. Johansson

**Affiliations:** US Center for Disease Control and Prevention (CDC), Dengue Branch, San Juan, Puerto Rico; Washington University in St Louis School of Medicine, UNITED STATES

## Abstract

Dengue viruses (DENV) are endemic in the US territories of Puerto Rico, American Samoa, and the US Virgin Islands, with focal outbreaks also reported in the states of Florida and Hawaii. However, little is known about the intensity of dengue virus transmission over time and how dengue viruses have shaped the level of immunity in these populations, despite the importance of understanding how and why levels of immunity against dengue may change over time. These changes need to be considered when responding to future outbreaks and enacting dengue management strategies, such as guiding vaccine deployment. We used catalytic models fitted to case surveillance data stratified by age from the ArboNET national arboviral surveillance system to reconstruct the history of recent dengue virus transmission in Puerto Rico, American Samoa, US Virgin Islands, Florida, Hawaii, and Guam. We estimated average annual transmission intensity (i.e., force of infection) of DENV between 2010 and 2019 and the level of seroprevalence by age group in each population. We compared models and found that assuming all reported cases are secondary infections generally fit the surveillance data better than assuming all cases are primary infections. Using the secondary case model, we found that force of infection was highly heterogeneous between jurisdictions and over time within jurisdictions, ranging from 0.00008 (95% CrI: 0.00002–0.0004) in Florida to 0.08 (95% CrI: 0.044–0.14) in American Samoa during the 2010–2019 period. For early 2020, we estimated that seropositivity in 10 year-olds ranged from 0.09% (0.02%–0.54%) in Florida to 56.3% (43.7%–69.3%) in American Samoa. In the absence of serological data, age-specific case notification data collected through routine surveillance combined with mathematical modeling are powerful tools to monitor arbovirus circulation, estimate the level of population immunity, and design dengue management strategies.

## Author summary

Viruses transmitted by *Aedes* mosquitoes are a substantial public health burden globally. In the US, the increasing number of outbreaks in recent decades and the co-circulation of the four dengue viruses present a risk of experiencing sequential infections, which can result in more severe disease. However, reported cases only represent a proportion of all infections, so the intensity of dengue circulation in US jurisdictions and the level of population immunity are largely unknown. Combining dengue surveillance data stratified by age with mathematical models enables direct estimation of transmission intensity and previous exposure. We found that dengue virus transmission intensity was highest in American Samoa and Puerto Rico. The US Virgin Islands also showed patterns of endemic transmission, while Hawaii and Guam present more sporadic outbreak profiles. These estimates can help improve risk assessment in these locations and guide interventions to the populations who most need them.

## Introduction

Dengue viruses (DENV) re-emerged in the Americas beginning in the 1970s, with the viruses rapidly expanding their range and associated burden of dengue infections [1–3]. However, this expansion has not been uniform. The four antigenically distinct dengue virus serotypes (DENV-1 to DENV-4) emerged at different times with affected areas experiencing different levels of transmission intensity, from sporadic outbreaks to endemic, year-round circulation [4–6].

The varied history of DENV transmission in the Americas directly shapes the current risk. Infection with one serotype generally provides long-term immunity to that serotype and short-term immunity to all serotypes. Secondary exposure to a different serotype from the primary infection has been associated with increased risk of severe dengue [7]. Population immunity is therefore modulated by the circulation of the four serotypes and the intensity of transmission of those viruses.

As population immunity and transmission intensity are intrinsically linked, it is challenging to estimate either of those components directly. Existing immunity can be partially measured with age-specific serological surveys, but these are logistically and financially challenging to implement, provide only a snapshot of previous exposure, and differentiating specific serotypes and the sequence of prior serotype exposures is very difficult. Transmission intensity can be characterized as the force of infection (FOI), the per capita rate at which susceptible individuals become infected by an infectious disease. A class of models called catalytic models can estimate the FOI, providing a measure of changes in exposure to diseases. However, direct estimation of the FOI is also challenging because population susceptibility is generally not known, many dengue infections are inapparent or unreported, and the proportion of individuals experiencing inapparent disease is dependent on prior infection [8–12]. Estimating the FOI therefore requires accounting for both underreporting and historical transmission intensity.

To help meet this challenge, age-stratified case data collected through passive surveillance can be used to gather insight into both existing immunity and transmission intensity [13]. Due to every age group having different exposure periods, incidence across each age group each year reflects both the age-group specific immunity and the yearly transmission intensity, enabling estimation of yearly transmission intensity for both current and past years [14]. Catalytic models that use age-specific case data can thus provide valuable insights into the dynamics of arboviruses [13,15,16] by quantifying infection burden in settings where seroprevalence data are unavailable or unrepresentative of the population.

**Fig 1:**
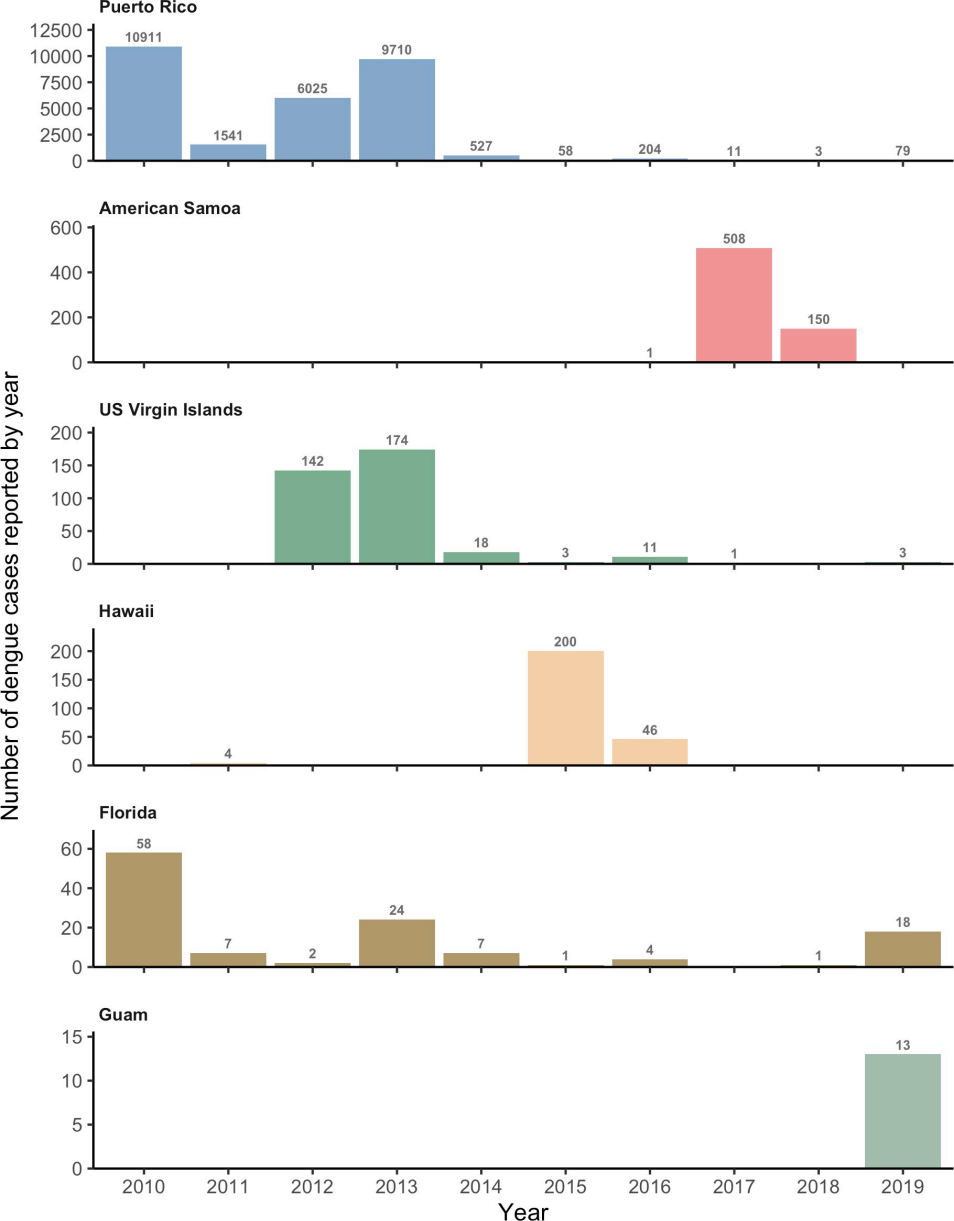
Confirmed and probable dengue cases reported to ArboNET by state or territory, 2010–2019. Included cases had no travel history and met laboratory criteria for diagnosis. Different y-axis scales were used for each jurisdiction.

Here, we analyze reported dengue case data from six US jurisdictions with outbreaks reported since dengue became a nationally reportable disease in 2010: Puerto Rico, American Samoa, US Virgin Islands, Florida, Hawaii and Guam. These locations represent diverse historical trends in reported case data, from long-term endemicity in Puerto Rico to no reported dengue in decades prior to an outbreak in 2019 in Guam (Fig 1). Building on previous work by Rodriguez-Barraquer et al. [13], we developed a generalized model that accounts for variability in transmission intensity and reporting over time and estimates the FOI and age-specific immunity over time. Accounting for yearly variability in FOI and reporting provides insights into dengue epidemiology in a wide variety of settings, from endemic to sporadic outbreaks, and can be used to better assess dengue burden in the US and other settings, contribute to epidemic risk assessment, and guide implementation and evaluation of control strategies such as vaccination.

## Methods

### Data sources

Dengue became a nationally notifiable disease in January 2010 and we analyzed confirmed and probable dengue cases reported to ArboNET for the years 2010–2019 for six jurisdictions: Puerto Rico, American Samoa, US Virgin Islands, Hawaii, Florida and Guam. Texas was excluded due to low case counts. During the analysis period, no dengue vaccines had been implemented in the United States. ArboNET includes information about place of likely acquisition (locally acquired or travel-associated) and disease severity. We analyzed locally acquired cases only and for all locations, we combined severe and non-severe cases. In Puerto Rico, where the highest number of severe cases was reported, we also developed a model fitting both cases and severe dengue cases simultaneously (including dengue hemorrhagic fever and dengue shock syndrome [17,18]). Age distribution data for each population was obtained from the 2010 census data provided by the United States Census Bureau (census.gov), except for Florida, Hawaii and Puerto Rico where in addition to the 2010 census data, population estimates were available for years 2011–2019. The ArboNET surveillance data is available to researchers upon request.

### Estimating the force of infection

We used a Bayesian catalytic model extending one developed by Rodriguez-Barraquer et al., [13] to estimate the time-dependent force of infection(FOI(t)), *λ*, of all circulating serotypes and derive age-specific seroprevalence estimates. Building on the original model, we (i) added random effects for both reporting and FOI probabilities to allow an overall average with yearly variability for each of these components. We also (ii) implemented separate models for different reporting possibilities (cases reported are primary DENV infections–Model P, secondary infections–Model S or a combination of both–Model PS) and (iii) accounted for potential infections in infants that appear more like secondary infections due to possible maternal antibody transfer. Finally, we (iv) generated a full suite of retrospective FOI estimates (prior to 2010, i.e., when ArboNET data became available).

The proportion of the population susceptible to the 4 dengue serotypes at time *t* and age group *a* declines with increasing age, at a constant rate *λ*:

$$S(a,t)=e^{-\int_{0}^{a}4\lambda (t-\tau )d\tau }.$$ (1)

*λ* is time-varying with a yearly time step. The FOI values presented are the sum of the 4 serotypes’ FOIs. For this analysis, serotype-specific data was not available, we therefore assumed here that the 4 serotypes have comparable transmission ability.

The proportion of individuals that already experienced a primary dengue infection, M, (for monotypic) in age group *a* and at time *t* is:

$$M(a,t)=4(e^{-\int_{0}^{a}3\lambda (t-\tau )d\tau })({1-e}^{-\int_{0}^{a}\lambda (t-\tau )d\tau }).$$ (2)

For the S model, the age-specific incidence of secondary infections, *I*_2_, for an individual who already experienced a monotypic infection is then the force of infection of the 3 other serotypes that individual can be infected with:

$$I_{2}(a,t)=3\lambda (t)M(a,t),$$ (3)

and the expected number of reported cases at time *t* for age *a*, is given by:

$$C(a,t)=I_{2}(a,t)r(t)P(a,t),$$ (4)

with r(t), the reporting probability varying over time and *P*(*a*, *t*), the population size of age group *a* at time *t*.

To account for the variability in the dataset, which may have some years with 0 reported cases as well as some with many cases, we allow for overdispersion of the number of cases reported using a negative binomial distribution:

Reportedcases(a,t)∼NB(C(a,t),ϕ), (5)

where *C*(*a*, *t*) is the total number of cases reported in age group *a* at time *t* and *ϕ* represents the overdispersion parameter in the negative binomial distribution. *ϕ* is computed as *ϕ* = 1/*ϕ*_*p*_^2^ with *ϕ*_*p*_~*Normal* (0, σ = 100).

#### Reporting probability

Many DENV infections are mild or asymptomatic, and do not result in a visit to a clinic or being reported as a case [8,9,19]. Additionally, multiple factors may lead to year-to-year fluctuation of reporting probabilities; the presence of other arboviruses infections may influence dengue reporting and testing, and, during epidemic years, doctors may be more likely to recognize and test dengue cases [20–23]. To allow some variability in reporting probabilities, we let it vary between years and partitioned the variance of the reporting prior distribution between uncertainty in the mean and uncertainty in year-to-year variability.

r0∼Normal(μ=μr,σ=σr22)logit(r(t))∼Normal(μ=r0,σ=σr22), (6)

where *r*_0_ is the average overall reporting parameter and *r*_*t*_, the random year effect. We used the logit transform so that we could implement the random effect with a normal distribution while maintaining a reporting probability between zero and one. Building on previous estimates [11,21,24] we set a prior distribution with median at 10% and 97.5^th^ percentile at 30% which translates into *μ*_*r*_ = -2.2 and *σ*_*r*_ = 0.7.

The probability of identifying and reporting dengue cases is also dependent on case severity. In one cohort study, the proportion of inapparent versus symptomatic case was similar between primary and secondary cases, with age, year, and time interval between consecutive infections more likely to affect symptomatic status [25]. Additionally, laboratory confirmation generally relies on virological testing that cannot differentiate primary and secondary infections, so the proportion of cases reported to ArboNET that are primary or secondary is not known. We therefore compared three different models considering either that all reported cases are secondary infections (Model S, described above), that reported cases are a combination of primary and secondary infections (Model PS), and that all reported cases are primary infections (Model P).

#### Model PS

For model PS, we assumed that primary and secondary infections are equally likely to be reported as a case. Equation for the age-specific incidence is then:

$$I_{1,2}(a,t)=4\lambda S(a,t)+3\lambda M(a,t).$$ (7)

#### Model P

The last model assumes that reported cases are exclusively primary infections. The age-specific incidence at age *a* and time *t* is then:

$$I_{1}(a,t)=4\lambda S(a,t).$$ (8)

Severe dengue cases, which are predominantly secondary infections, are also reported to ArboNET. Because substantial numbers of severe cases were reported in Puerto Rico during the study time period, we performed a secondary analysis for Puerto Rico fitting the model to cases and severe dengue cases, simultaneously. While severe cases are more likely to be reported than non-severe cases, severe cases represent only a small proportion of secondary infections. In previous cohort studies, the estimated probability of reporting a severe case among secondary infections was below 5% (less than 1% [25] and 3% [26]). Based on those studies, we fitted severe dengue cases as being a fraction of all reported dengue cases and used a beta prior distribution with *α* = 2 and *β* = 1. From this fraction of cases reported as severe among all cases, we were able to estimate the reporting probabilities of severe cases among secondary cases.

#### FOI

The FOI is likely to vary over time depending on the circulating dengue serotypes, serotype introduction or re-introduction, and population immunity, but few catalytic models account for this fluctuation [27]. Similar to the reporting probabilities (described above), we introduced a hierarchical structure in the model for the FOI:

λ0∼Normal(μ=μλ,σ=σλ22)logit(λt(t))∼Normal(μ=λ0,σ=σλ22), (9)

where *λ*_0_ is the overall average FOI and *λ*_*t*_, the random year effect. To use better informed priors, we estimated a specific *μ*_*λ*_ and *σ*_*λ*_ prior distribution for each location, using the average age of infection, life expectancy, and the mean reporting prior probability. If we consider ℛ_0_≃1+*L*/*A*, ℛ_0_ being the basic reproduction number in a fully susceptible population, *L*, the average life expectancy and *A*, the age at first infection [28] and consider the susceptible population, *S*, as being the inverse of ℛ_0_, we can derive the average FOI as the mean incidence divided by *S*. The mean incidence being the average yearly number of cases in the population and the average life expectancy in the US set to 77 years [29]. Finally, we retrieve *μ*_*λ*_ and *σ*_*λ*_ by using the logitnorm function in R parameterized with average FOI as the median and 97.5^th^ percentile at 20%.

The FOI priors used for each location are available in Table A (S1 Document).

#### Infant cases

The presence of maternal antibodies may interfere and potentially predispose some infants to more severe disease, such that infant infections may be more severe and more likely to be reported than expected for a true primary infection [30]. To account for this, we added a proportion parameter, *α*, that sets the initial previous exposure for infants to a proportion of the FOI in the previous year (i.e., through their mother’s exposure) rather than zero (see Figs A and O in S1 Document). The force of infection experienced by individuals in the first age group (i.e., under one-year olds) is then calculated as *α* times the force of infection that individuals experienced the previous year.

This is implemented in all three models S, P and PS.

#### Model comparison

Models were compared using leave-one-out (loo) Pareto smoothed importance sampling [31]. Lower loo scores indicate better cross-validation model fit. We also collected yearly log-likelihood estimates to assess how our S, P and PS models fit to the reported case data. For each parameter we extracted the median and 2.5% and 97.5% percentiles of the posterior distribution to obtain the 95% Bayesian credible intervals (hereafter 95% CrI). Models were run using Bayesian Markov Chain Monte Carlo sampling with a No-U-Turn sampler using the RStan package [32] in R (version 4.1.2, [33]).

For each location, we fitted the 3 models to the ArboNET case notification data available from 2010 to 2019 for individuals aged 0 to 80 years old and ran the Bayesian model on 4 chains for 50,000 iterations, with each chain performing 25,000 burn-in steps and a thinning every 10 steps. We visually inspected chains to ensure stationarity and evaluated model convergence using $\hat{R}$ and *n*_*eff*_/*N* ratios statistics [32]. We checked that all parameters where identifiable by comparing prior and posterior distributions (see Fig E in S1 Document). Code to implement the model is available on GitHub at https://github.com/sarahkada/dengue-catalytic-model.

## Results

**Fig 2:**
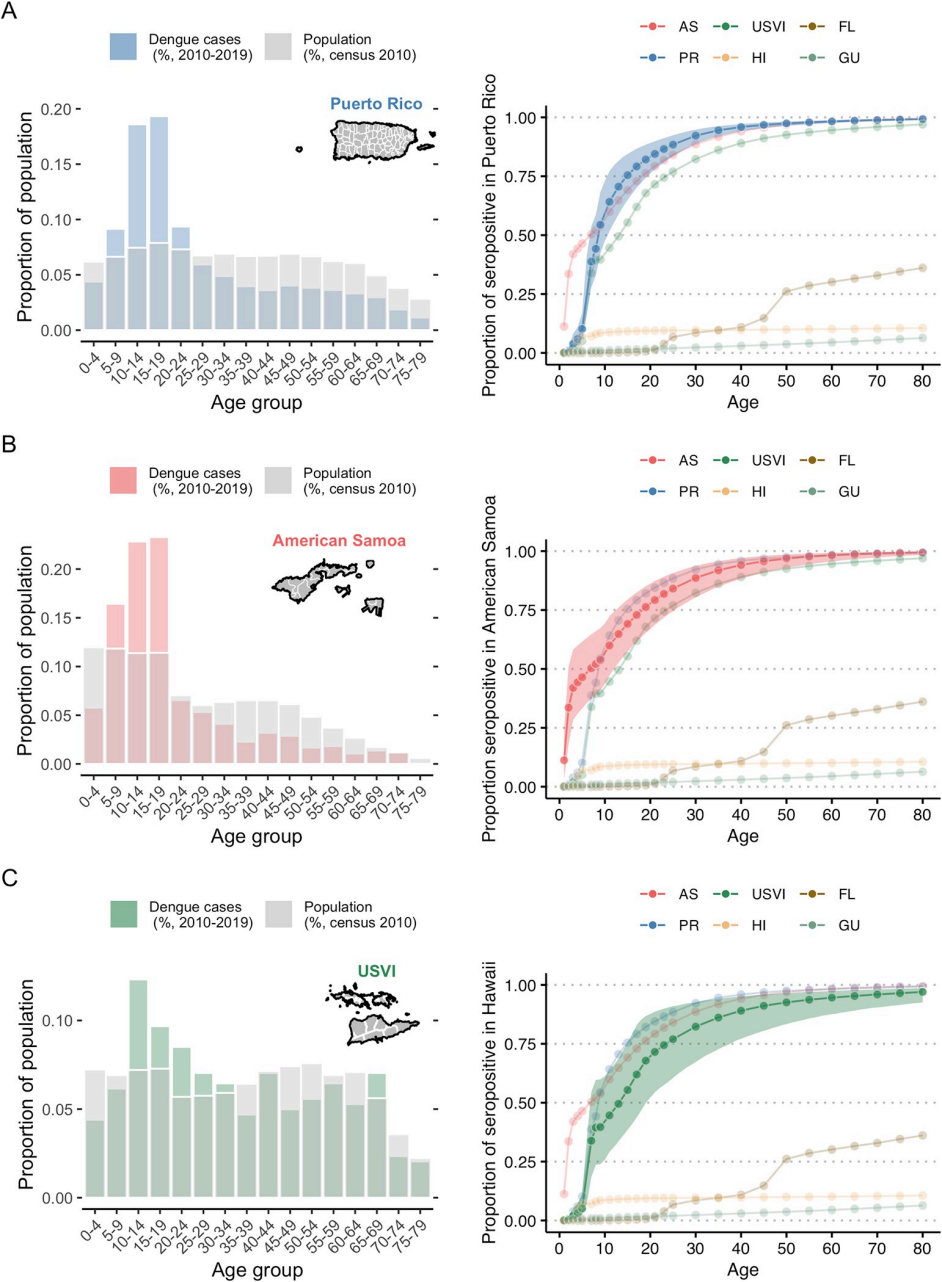
Age distribution of reported dengue cases and 2019 seroprevalence estimates by age group for each US state and territory assessed. Age distribution of all locally reported, confirmed and probable dengue cases in Puerto Rico (PR, A), American Samoa (AS, B) and US Virgin Islands (USVI, C) in 2010–2019 (colored bar) and age distribution of the population in these respective territories (grey bars, 2010 census) (left panel) and age distribution of 2019 seroprevalence estimate obtained from the fit of model S (reported cases are secondary infections only), with corresponding 95% credible intervals (shaded area, right panel). Detailed tables on years with reported dengue cases are available in S1 Document. Y-axis scale on the left panels differ for each location. Base layers for Puerto Rico were retrieved from the US Census Bureau TIGER/Line files [56]. American Samoa and US Virgin Islands shapefiles were obtained from GADM (https://gadm.org/). Terms and conditions of use available from https://gadm.org/license.html.

**Fig 3:**
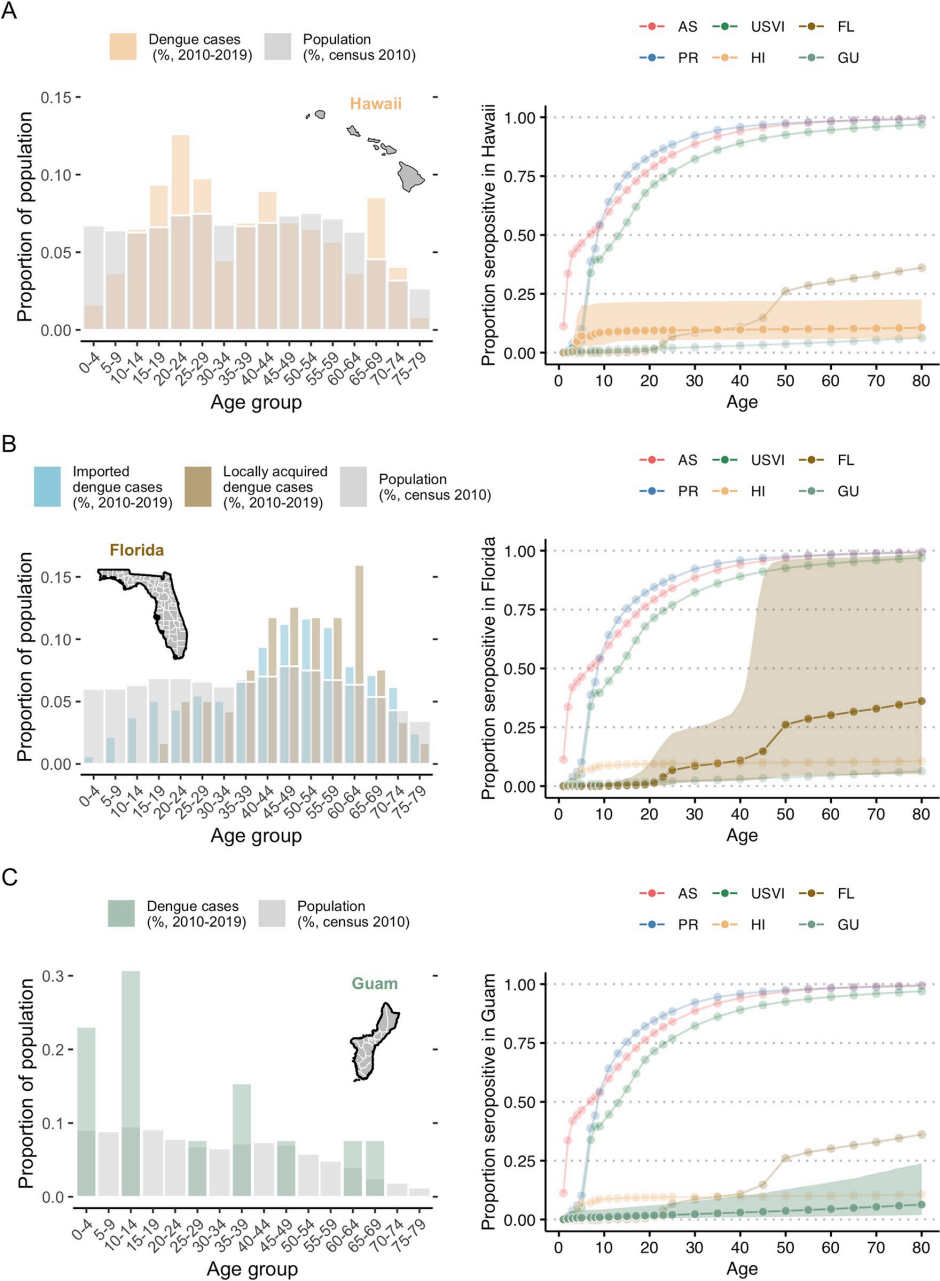
Age distribution of reported dengue cases and 2019 seroprevalence estimates by age group for each US state and territory assessed. Age distribution of all locally reported, confirmed and probable dengue cases in Hawaii (HI, A), Florida (FL, B) and Guam (GU, C) in 2010–2019 (colored bar) and age distribution of the population in these respective territories (grey bars, 2010 census) (left panel) and age distribution of 2019 seroprevalence estimate obtained from the fit of model S (reported cases are secondary infections only), with corresponding 95% credible intervals (shaded area, right panel). Florida contains the additional age distribution of imported cases. Detailed tables on years with reported dengue cases are available in S1 Document. Y-axis scale on the left panels differ for each location. Map shapefiles for Hawaii and Florida were obtained from the *urbnmapr* [57] and *maps* [58] R packages, respectively. Base layers for Guam were retrieved from the US Census Bureau TIGER/Line files [59].

Dengue circulation was highly heterogeneous during 2010–2019 across the six jurisdictions (Fig 1). The age distribution of cases also varied markedly, with the most commonly reported age group being 10–14 years in the US Virgin Islands and Guam to 60–64 years for locally acquired cases in Florida (Figs 2 and 3). If all age groups had the same risk of infection, the age distribution of cases would mirror the population age distribution, e.g., the largest age group is more likely to have the highest number of reported cases. However, immunity from prior infection, especially in endemic areas, shifts the age of cases to predominantly younger age groups (e.g., Puerto Rico, American Samoa).

**Fig 4:**
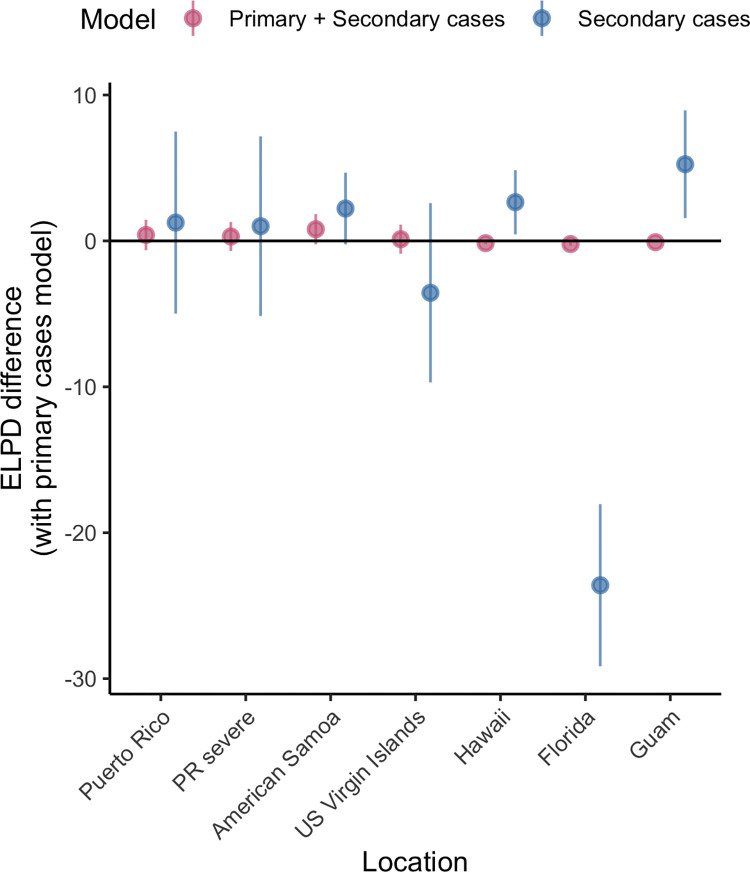
Leave-one-out cross validation model comparison using the estimated difference in ELPD (expected log pointwise predictive probabilities). ELPD is a measure of out-of-sample predictive accuracy, as estimated by the Bayesian leave-one-out cross validation (LOO). We compared model P (cases as primary) to models PS and S (primary and secondary cases and secondary cases only). Models under the horizontal bar at 0 fit the data better than model P (reference). Vertical bars represent the corresponding standard error of the difference in ELPD.

We fitted models to each time series of age-specific case data under three different assumptions: (i) that all reported cases are primary infections (Model P), (ii) that all reported cases are secondary infections (Model S), and (iii) that reported cases include both primary and secondary cases (Model PS). In Hawaii and Guam, Model P and Model PS had significantly lower loo error than Model S (Fig 4). The fit of Model S was only significantly better for local cases in Florida (i.e., excluding travel-associated cases) though also had lower mean error for the US Virgin Islands and the model simultaneously fitting to severe cases in Puerto Rico.

Comparing the different models across years, age groups and locations, we found mixed evidence about relative fit. For Puerto Rico, log likelihoods indicated that Model S tended to have a better fit in years with more reported cases and sometimes a worse fit in years and age groups with fewer reported cases (Figs F-G in S1 Document). Model S also appeared to fit the distribution of cases across younger age groups more accurately across most jurisdictions (Figs H-N in S1 Document). Models P and PS both tend to estimate that a larger number of cases should be reported in the youngest age group (0–4 years old). Model S qualitatively better matches the distinct pattern of dengue cases in children across endemic areas. Because of this distinct advantage and the finding that fit for Model S was only inferior for Hawaii and Guam (where there was the least evidence of sustained transmission) (Figs H-N in S1 Document), we used Model S for subsequent analyses.

**Fig 5:**
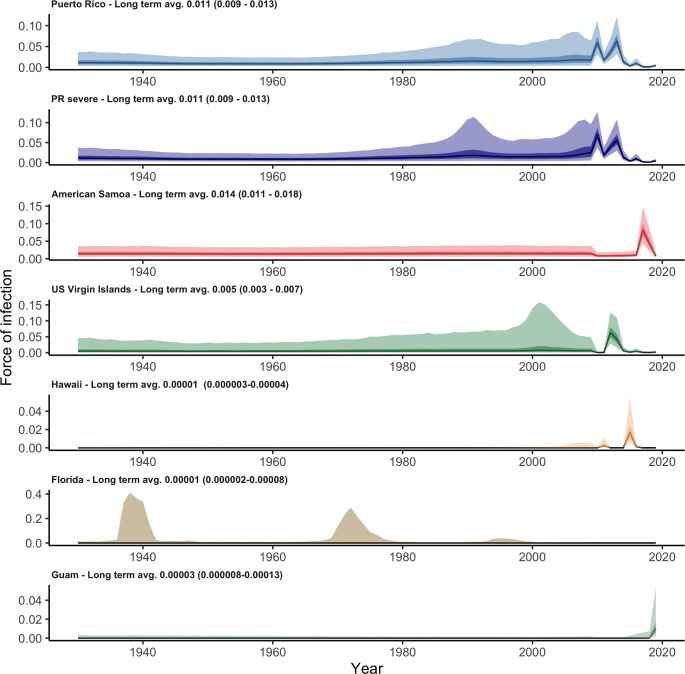
Yearly estimates of the force of infection (FOI) in Puerto Rico (all cases) and model combining severe cases (PR severe), American Samoa, US Virgin Islands, Hawaii, Florida and Guam in 2010–2019 from our model S (cases as secondary infections). The numbers in the upper left-hand corner of each panel shows overall average FOI estimates for each location. Dark and light shaded areas represent respectively, 50% and 95% credible intervals. Different y-axis scales were used for each jurisdiction.

**Fig 6:**
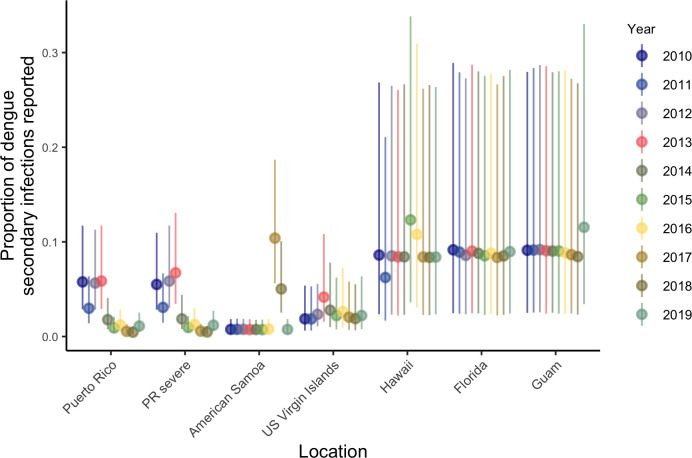
Estimates of the yearly probability of reporting cases in Puerto Rico (all cases) and model combining all cases and severe cases (PR severe), American Samoa, US Virgin Islands, Hawaii, Florida and Guam in 2010–2019 from our S model (cases are considered secondary infections). Vertical bars represent 95% credible intervals.

**Fig 7:**
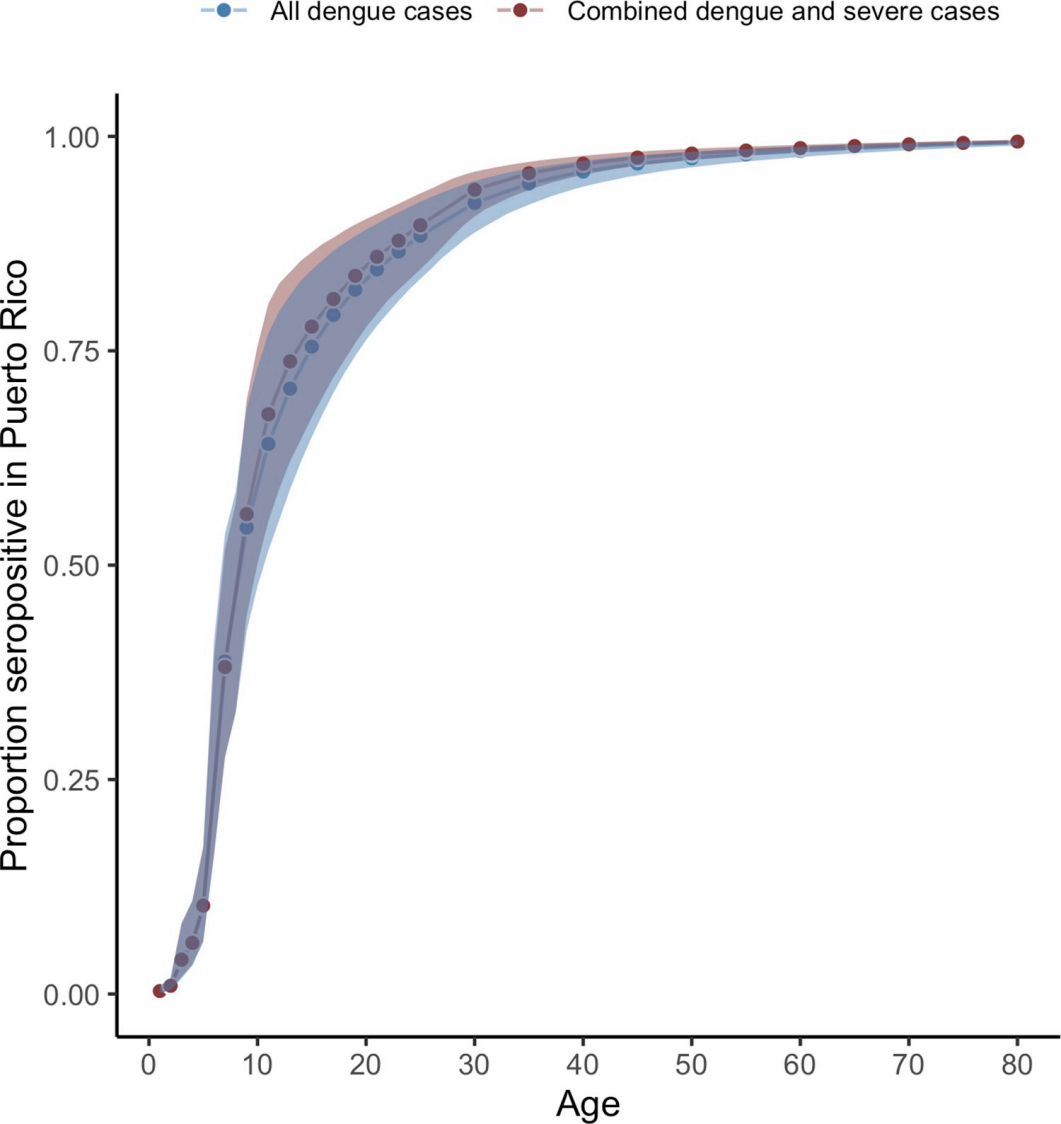
Age-specific seropositivity estimates for 2019 in Puerto Rico using all dengue cases and the model fitted to both dengue cases and severe dengue cases. Derived from model S where reported cases are secondary infections only. Shaded areas represent 95% credible intervals.

Of the six locations analyzed, Puerto Rico had the second highest overall average FOI (parameter *λ*_0_), estimated at 1.1% (95% CrI: 0.9–1.3%) with substantial year-to-year variation including higher FOI estimates in the early 1990s and 2000s (prior to the dataset analyzed here, Fig 5). The estimated probability of a secondary infection resulting in a reported dengue case was 2.1% (95% CrI: 1.4–3.3%). The estimated fraction of severe cases among all dengue cases was 1.9% (95% CrI: 1.6–2.2%). Reporting was estimated to be slightly higher in years when more cases were reported (Fig 6). For 2019, we estimated seroprevalence in 5-year-old children to be 10.1% (95% CrI: 6.1–16.9%), increasing to 94.5% (95% CrI: 92–96.2%) by age 35 (Fig 2A). FOI and seroprevalence estimates using severe cases were similar to those using all cases in Puerto Rico (Fig 7). However, we observed higher uncertainty in the FOI estimates in the 1990s in the model using severe cases, potentially indicating the existence of an outbreak (Fig 5).

While no cases were reported in American Samoa between 2010 and 2016, the model estimated low reporting in 2010–2016 (Fig 6) and high uncertainty for FOI prior to 2010 with the highest overall average FOI of all locations at 1.4% (95% CrI: 1.1–1.8%, Fig 5). The high estimated FOI in 2017–2018 translated to an estimated 2019 seroprevalence in 2019 for 5-year-olds of 46.5% (95% CrI: 33.2–61.6%, Fig 2B). Thus, the age structure of cases in 2017–2018, indicates that while this was a large outbreak, there was likely dengue transmission both in 2010–2016 and in preceding years. The FOI estimate for American Samoa was higher than the estimate for Puerto Rico, with similar estimated adult seroprevalence (91.9%, 95% CrI: 84.8–95.7% for 35-year-olds, Fig 2B).

The US Virgin Islands had lower estimated overall average FOI (0.5%, 95% CrI: 0.3–0.7%, Fig 5) and more stable estimated reporting (Fig 6). With a low FOI in recent years, the 5-year-old estimated seroprevalence for 2019 was low (5.1%, 95% CrI: 2.3–11%, Fig 2C) but cumulative exposure in adults was still high, with 35-year-old estimated seroprevalence for 2019 of 86.1% (95% CrI: 72.7–94.1%, Fig 2C).

Hawaii reported two outbreaks (2011 and 2014–2015) and Guam reported one (2019) during the study period (Fig 1) and both had less distinct age-specific case patterns (Fig 3A and 3C). The estimates for reporting largely reflected the reporting prior distribution (Fig 5), indicating that there was little information between the case numbers and case age data to distinguish differences in reporting versus FOI. Nonetheless, overall average FOI estimates were low for both jurisdictions (Hawaii: 0.001%, 95% CrI: 0.0003–0.004%; Guam: 0.003%, 95% CrI: 0.0008–0.013%; Fig 5), with significant increases in the years when the outbreaks were reported. The 2019 seroprevalence estimates were low for all age groups in both locations: 6.3% (95% CrI: 3.9–12.3%, Fig 3A) and 2.6% (95% CrI: 0.8–8.9%, Fig 3C) for 35-year-olds in Hawaii and Guam, respectively.

Florida was unique in that 90.5% of cases had reported travel histories. To assess local transmission within the state, we therefore only analyzed cases without reported travel (Fig 3B). Local dengue cases were reported in multiple years and had a distinctly different age profile for cases compared to other locations, with no reported cases in younger age groups and many among adults, which resulted in the largest uncertainty for seroprevalence estimates, especially for older age groups (Fig 3B). These estimates however indicated confidence in lower seroprevalence for younger adults, 9.6% (95% CrI: 2.0–28.1%) for 35-year-olds. This low seroprevalence estimate for local cases indicates a relatively low historical transmission intensity locally, implying that many imported secondary cases likely also had their primary infections outside of Florida. The overall average FOI for Florida considering only local cases was 0.001% (95% CrI:(0.0002–0.008%). Similar to Hawaii and Guam, there was insufficient information to further resolve the probability of reporting a case and the estimated reporting probabilities resembled the prior.

Across the six locations, estimated overall average FOI was highest for American Samoa and Puerto Rico, followed by the US Virgin Islands, with much lower estimates for Hawaii and Guam. In addition to the computation of the overall average FOI, we also calculated a long-term average FOI by taking the average of yearly FOI values over two periods: 1929–2009 and 2010–2019. We found that for the higher FOI locations (Puerto Rico, American Samoa, and US Virgin Islands), the yearly average was slightly lower for the period which included surveillance data (2010–2019) compared to estimates for time periods prior to 2010, for which ArboNET data are not available. In contrast, the estimated FOIs for the 2010–2019 period were considerably higher than the historical period for Hawaii and Guam.

## Discussion

Passive surveillance is the most common approach for dengue surveillance globally [34–36]. While passive surveillance can be highly effective for monitoring longitudinal changes in incidence, insight into disease burden is challenging as care seeking behavior, access to care, case definitions, reporting, and resources can differ substantially over time and across jurisdictions. Differences in these components can mean that actual increases, decreases, or jurisdictional differences in dengue burden are difficult to disentangle from reporting dynamics. The importance of understanding dengue burden beyond reported incidence has grown in recent years, with the advent of dengue vaccines which can be used to reduce the burden, but also require consideration of levels of preexisting population immunity for effective deployment [37]. Here, we extended a previously developed Bayesian model to leverage the age distribution among cases reported via passive surveillance in the US to estimate yearly DENV force of infection, yearly reporting probabilities, and seroprevalence for six jurisdictions with very different histories of reported dengue incidence.

Across the six jurisdictions, the estimated dengue burden was highly heterogeneous and FOI estimates did not appear to be higher over time, except in Hawaii and Guam. We found evidence of high long-term transmission intensity in Puerto Rico, American Samoa, and US Virgin Islands (FOI and high seroprevalence in adults), despite different histories of dengue in each location. Hawaii, Florida, and Guam also differed in the frequency and magnitude of reported outbreaks, but all had considerably lower long-term FOIs.

The higher estimated FOIs in Puerto Rico, American Samoa, and US Virgin Islands reflect different dynamics. Puerto Rico has consistently reported dengue cases for at least three decades ([38,39], timeline by [6]), while dengue reporting in American Samoa and the US Virgin Islands is more sporadic. Outbreaks have been reported in American Samoa in the 1970s, 1990s, 2000s, and 2017–2018 [40–42] and in the US Virgin Islands in the 1990s, 2000s, and in 2012–2013 [6,43]. Here, we found that despite these different apparent dynamics, the estimated overall average FOI and seroprevalence in these locations were similar, with a high likelihood that individuals experienced a secondary infection by the time they reached 10 years old (median seroprevalence estimates at 42% and 59% in the US Virgin Islands and Puerto Rico, respectively). Three seroprevalence studies support these findings. First, a study in the municipality of Patillas in the southeastern part of Puerto Rico [44] found a 42.5% seroprevalence in 10–11 year-olds in 2007–2008. For 10-year-olds, we estimated a seroprevalence of 57.2% (95% CrI: 45.3–69.6%) in 2010—the earliest estimate available for our study—in Puerto Rico. A 2010 seroprevalence study in American Samoa found a seroprevalence of 95.6% (95% CrI 93.9–96.8%) for 18–87 year-olds, with slightly lower estimates for 18–25 year-olds (89.1%, 95% CrI: 84.0–92.6%) [45]. For the same age groups and year, using only passive surveillance data, we estimated a seroprevalence of 95.9% (95% CrI: 86.9–98.5%) and 86.9% (95% CrI: 63.6–95.6%) for these respective age groups. More recently, CDC’s updated traveler risk classifications re-categorized US Virgin Islands from “Sporadic/Uncertain” to “Frequent/Continuous” [46]. Additionally, a cross-sectional seroprevalence study conducted in US Virgin Island schools in April 2022 estimated a seroprevalence of 42% (95% CrI: 26–60%) and 54% (95% CrI: 18–89%) among 10 and 13 year-olds respectively [47], while our model estimated a seroprevalence of 34% (95% CrI: 18–54%) and 41% (95% CrI: 26–61%) in 7 and 13 year-olds for 2019, 3 years prior. No major outbreak has been reported in the US Virgin Islands between 2019 and 2022. The high level of immunity found in the US Virgin Islands with our model confirms that dengue transmission intensity in this territory is likely higher than previously suspected.

In Hawaii, low FOI and seroprevalence estimates are consistent with several possible histories: small intermittent outbreaks, rare large epidemics, or continuous low intensity transmission. No autochthonous cases had been reported in Hawaii between 1944 and 2001, but recent outbreaks were reported in 2001–2002 [48], 2011, and 2015–2016 [49]. Given some reporting issues during these outbreaks (see [50]), the seroprevalence profile in Hawaii should be interpreted with caution. Although the results here do not differentiate between the possible histories, they do indicate that substantial and sustained undetected transmission in recent decades is highly unlikely. Further serological data collection could help decipher between these hypotheses.

In Florida, we found a low FOI and a low level of immunity, suggesting low circulation of the virus. Frequent introductions of dengue viruses by travelers result in frequent opportunities for local transmission, so FOI estimates likely reflect both this relatively high introduction risk and limited local transmission. We can hypothesize that the travel and migration history of Florida residents may also contribute to a higher seroprevalence among older individuals than what would be expected from local transmission alone. In Miami-Dade County where the majority of dengue cases in Florida have been reported, the U.S. Census estimates that 54% of the population was born in a foreign country [51]. Added together, these risk factors could explain the higher case numbers in older adults, high uncertainty in seroprevalence estimates for those adults, and the better fit of the secondary case model to the Florida data. More detailed data on histories of travel and dengue exposure could potentially provide more resolution on historical patterns of transmission in Florida as compared to exposures acquired elsewhere that impact the patterns of dengue seen in Florida today.

Our FOI and seroprevalence estimates in Guam were lower than for the other US territories with comparable environmental conditions. According to our model, primary infections, or a combination of primary and secondary infections dominated the 2019 outbreak in Guam, which is consistent with other studies that found no evidence of endemic circulation in Guam since World War II [42,52,53], potentially due to successful vector control strategies limiting dengue transmission on this territory [52].

Our model estimated time-varying reporting probabilities for all jurisdictions and found important variations across locations and years. In locations with lower overall average FOI, there was little information in the case data to update the broad prior distribution’s assumption for reporting probabilities of approximately 10–30%. However, for locations with higher overall average FOI, reporting was generally below 10% and showed substantial year-to-year variability. Other recent work indicates the possibility of similar variation between years [21,23] and particularly between epidemic and non-epidemic years [22], a pattern which we also found in the data and models analyzed here. These findings indicate the likely importance of accounting for variability in reporting probabilities over time.

We compared models accounting for reported cases as primary, secondary, or a combination of both primary and secondary infections, finding mixed evidence of fit across jurisdictions. For Florida, the secondary case model clearly fit better by leave-one-out metrics, while it was worse than the other models for Hawaii and Guam (Fig 4). However, detailed examination of fitted model estimates and log-likelihoods revealed that models for secondary infections provided better qualitative fits to the age profiles of cases in locations with higher seroprevalence and for years and age groups with higher incidence. Thus, despite equivocal out-of-sample performance, we found that the secondary-only model is likely preferrable in locations with high FOI or with high seroprevalence that may have been acquired elsewhere (Florida), while the primary or combined models may perform better in low FOI settings with limited previous exposure. For reporting, this finding implies that most cases reported in Puerto Rico, American Samoa, US Virgin Islands and Florida are secondary infections and the majority of reported cases in Hawaii and Guam are likely primary infections. This confirms that in endemic settings primary infections are largely unreported, but also suggests this pattern is slightly different in locations with more sporadic transmission. This spatial and temporal variation in reporting is likely to be a more general phenomenon.

While we were able to identify clear differences in transmission intensity across locations, this analysis provides limited insight into why those differences exist. Different levels of mobility and interactions with mosquitos between individuals within and between households likely lead to some differences. Environmental variables (e.g., temperature and rainfall) also impact the mosquito population and may drive differences in transmission intensity [54,55]. Furthermore, we accounted for previous infections as a condition for reporting and a condition for the force of infection. However, we did not consider how heterotypic and homologous immunity to different serotypes (or other flaviviruses) may substantially impact these dynamics.

Some limitations are worth noting. First, ArboNET includes dengue data since 2010 but in some jurisdictions few cases have been reported in this period, meaning that for some jurisdictions the models rely on relatively few data. While fitting the data in a Bayesian framework with informative priors reduces the possibility of misleading conclusions, high uncertainty may remain around some important parameters (e.g., reporting in some jurisdictions). Heterogeneity in dengue exposure and disease across age and gender may result in both age-specific FOI and age-specific reporting probabilities. Neither of these possibilities are captured in our model implementation and would be difficult to assess without informative priors for that age-specific variation. In early model development, we found that to account for time-variable reporting and FOI, we needed to have informative priors for both components. Another possible area for advancing this type of model would be to further differentiate reporting probabilities for primary and secondary infections (see [15]). With informative priors, this could potentially provide further resolution on location specific differences in transmission where reported cases may be dominated by primary infections, secondary infections, or a mix.

Our study shows that analyzing age-specific case notification data with catalytic models can provide invaluable insight into dengue virus transmission dynamics beyond simple case counts, including estimates of transmission intensity over time and of evolving population-level exposure (as an ongoing proxy for seroprevalence). These components can help identify where interventions such as dengue vaccines should be prioritized in the US and elsewhere or what populations may be at higher risk of secondary infections and therefore severe disease. These models may provide an important tool for assessing and monitoring dengue transmission risk in many locations where age-specific surveillance data already exist.

## Disclaimer

The findings and conclusions in this report are those of the authors and do not necessarily represent the official position of the Centers for Disease Control and Prevention.

## Supporting information

S1 Document Table A. Values for the mean and sigma values of reporting and FOI used in each location. The same reporting priors were used across all locations. **Table B. Force of infection estimates in Puerto Rico, American Samoa, US Virgin Islands, Hawaii, Florida and Guam from 2010 to 2019.** Median and 95% Bayesian credible intervals. **Table C. Long-term average of force of infection estimates in Puerto Rico, American Samoa, US Virgin Islands, Hawaii, Florida and Guam for the 1929–2009 and 2010–2019 time periods.** The 2010–2019 period corresponds to the period with data. **Fig A. Prior distribution of the reporting hyperprior.** Dashed lines represent median prior value, at 10%. **Fig B. Age distribution of reported severe and non-severe dengue cases (colored bars) in Puerto Rico from 2010 to 2019.** The grey bars represent the age distribution of the population (US census, 2010). **Fig C. Yearly age distribution of dengue reported cases in Puerto Rico (A), American Samoa (B), US Virgin Islands (USVI, C), Hawaii (D), Florida (E), and Guam (F).** Scales may be adjusted for years with fewer dengue cases reported. **Fig D. Yearly age distribution of locally-acquired and imported dengue reported cases in Florida.** Scales are adjusted for years with fewer dengue cases reported. **Fig E. Violin plots of FOI and reporting probability prior and posterior estimates in Puerto Rico (A), American Samoa (B), the US Virgin Islands (C), Hawaii (D), Florida (E) and Guam (F) from 2010 to 2019.** Points represent the median value of each estimate and the dashed lines represent the long term average of FOI and reporting probability estimates. **Fig F. Comparison of log likelihood samples by age group and years of models Primary, Primary & Secondary, and Secondary using Puerto Rico data.** Models’s log-likelihood values may overlap. The log likelihood value in a model is a measure of goodness of fit. The higher the value (i.e., closer to 0), the better. **Fig G. Heatmap comparison between models Primary, Primary and Secondary, and Secondary of log likelihood median samples and cases by age group and years using Puerto Rico data.** The log likelihood value in a model is a measure of goodness of fit. Here, models with log likelihood values closest to 0 were plotted. **Fig H. Reported cases model fit (all cases and severe cases only) by age group in Puerto Rico from 2010 to 2019.** Points represent cases reported to ArboNET while lines represent model fit to Primary, Primary & Secondary, and Secondary models. Shaded areas represent the best model 95% CrI. **Fig I. Reported cases model fit to model fitted to all dengue (A) and severe cases (B) by age group in Puerto Rico from 2010 to 2019.** Points represent dengue (A) and severe cases (B) reported to ArboNET while lines represent model fit to Primary, Primary & Secondary, and Secondary models. Shaded areas represent the best model 95% CrI. **Fig J. Reported cases model fit by age group in American Samoa from 2010 to 2019.** Points represent cases reported to ArboNET while lines represent model fit to Primary, Primary & Secondary, and Secondary models. Shaded areas represent the best model 95% CrI. **Fig K. Reported cases model fit by age group in US Virgin Islands from 2010 to 2019**. Points represent cases reported to ArboNET while lines represent model fit to Primary, Primary & Secondary, and Secondary models. Shaded areas represent the best model 95% CrI. **Fig L. Reported cases model fit by age group in Hawaii from 2010 to 2019.** Points represent cases reported to ArboNET while lines represent model fit to Primary, Primary & Secondary, and Secondary models. Shaded areas represent the best model 95% CrI. **Fig M. Reported cases model fit by age group in Florida from 2010 to 2019.** Points represent cases reported to ArboNET while lines represent model fit to Primary, Primary & Secondary, and Secondary models. Shaded areas represent the best model 95% CrI. **Fig N. Reported cases model fit by age group in Guam from 2010 to 2019.** Points represent cases reported to ArboNET while lines represent model fit to Primary, Primary & Secondary, and Secondary models. Shaded areas represent the best model 95% CrI. **Fig O. Alpha parameter posterior estimates with 95% CrI (vertical bars) in Puerto Rico, Puerto Rico severe (using severe cases only), American Samoa, US Virgin Islands, Hawaii, Florida and Guam.**
(PDF)
