# Supplementary material for: Age-specific case data reveal varying dengue transmission intensity in US states and territories
Source: PLoS Negl Trop Dis. 2024 Mar 1;18(3):e0011143. doi: 10.1371/journal.pntd.0011143 (PMC10936865; doi:10.1371/journal.pntd.0011143)
Supplement: S1 Document — The same reporting priors were used across all locations. Table B. Force of infection estimates in Puerto Rico, American Samoa, US Virgin Islands, Hawaii, Florida and Guam from 2010 to 2019. Median and 95% Bayesian credible intervals. Table C. Long-term average of force of infection estimates in Puerto Rico, American Samoa, US Virgin Islands, Hawaii, Florida and Guam for the 1929–2009 and 2010–2019 time periods. The 2010–2019 period corresponds to the period with data. Fig A. Prior distribution of the reporting hyperprior. Dashed lines represent median prior value, at 10%. Fig B. Age distribution of reported severe and non-severe dengue cases (colored bars) in Puerto Rico from 2010 to 2019. The grey bars represent the age distribution of the population (US census, 2010). Fig C. Yearly age distribution of dengue reported cases in Puerto Rico (A), American Samoa (B), US Virgin Islands (USVI, C), Hawaii (D), Florida (E), and Guam (F). Scales may be adjusted for years with fewer dengue cases reported. Fig D. Yearly age distribution of locally-acquired and imported dengue reported cases in Florida. Scales are adjusted for years with fewer dengue cases reported. Fig E. Violin plots of FOI and reporting probability prior and posterior estimates in Puerto Rico (A), American Samoa (B), the US Virgin Islands (C), Hawaii (D), Florida (E) and Guam (F) from 2010 to 2019. Points represent the median value of each estimate and the dashed lines represent the long term average of FOI and reporting probability estimates. Fig F. Comparison of log likelihood samples by age group and years of models Primary, Primary & Secondary, and Secondary using Puerto Rico data. Models’s log-likelihood values may overlap. The log likelihood value in a model is a measure of goodness of fit. The higher the value (i.e., closer to 0), the better. Fig G. Heatmap comparison between models Primary, Primary and Secondary, and Secondary of log likelihood median samples and cases by age group and years using [file pntd.0011143.s001.pdf]

Supporting Information

Age-specific case data reveal varying dengue transmission intensity in US states and territories

Sarah Kada[1]*, Gabriela Paz-Bailey[1], Laura E. Adams[1] & Michael A. Johansson[1]

[1]US Center for Disease Control and Prevention, Dengue Branch, San Juan, Puerto Rico

*Corresponding author: sarahkada@yahoo.fr

Contents

1 Model specifications **1**
1.1 Choice of priors 1

2 Supplementary figures **3**
2.1 Age distribution of cases 3
2.2 Parameter identifiability 8
2.3 Model comparison using log likelihood 15
2.4 Fit to reported cases 19
2.5 Other parameters 25

1 Model specifications

1.1 Choice of priors

Using the median and upper quantile priors, we retrieve the parameters of the inverse logitnormal distribution with the R package **logitnorm** (version 0.8.38, Wutzler, 2021).

Table A. Values for the mean and sigma values of reporting and FOI used in each location. The same reporting priors were used across all locations.

	μ	σ_r	σ_λ
Reporting, r	- 2.2	0.7	
FOI, λ (Puerto Rico)	- 3.12		0.88
FOI, λ (Puerto Rico, combined with severe cases)	- 3.12		0.88
FOI, λ (American Samoa)	- 2.68		0.66
FOI, λ (US Virgin Islands)	- 4.53		1.6
FOI, λ (Hawaii)	- 7.4		3.09
FOI, λ (Florida)	- 10.99		4.9
FOI, λ (Guam)	- 7.44		3.09

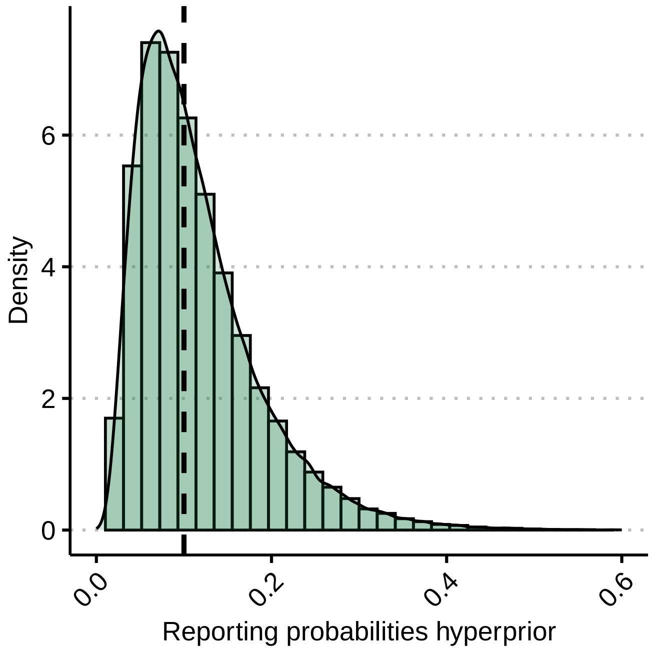

Fig A. Prior distribution of the reporting probability hyperprior. Dashed lines represent median prior value, at 10%.

2 Supplementary figures

2.1 Age distribution of cases

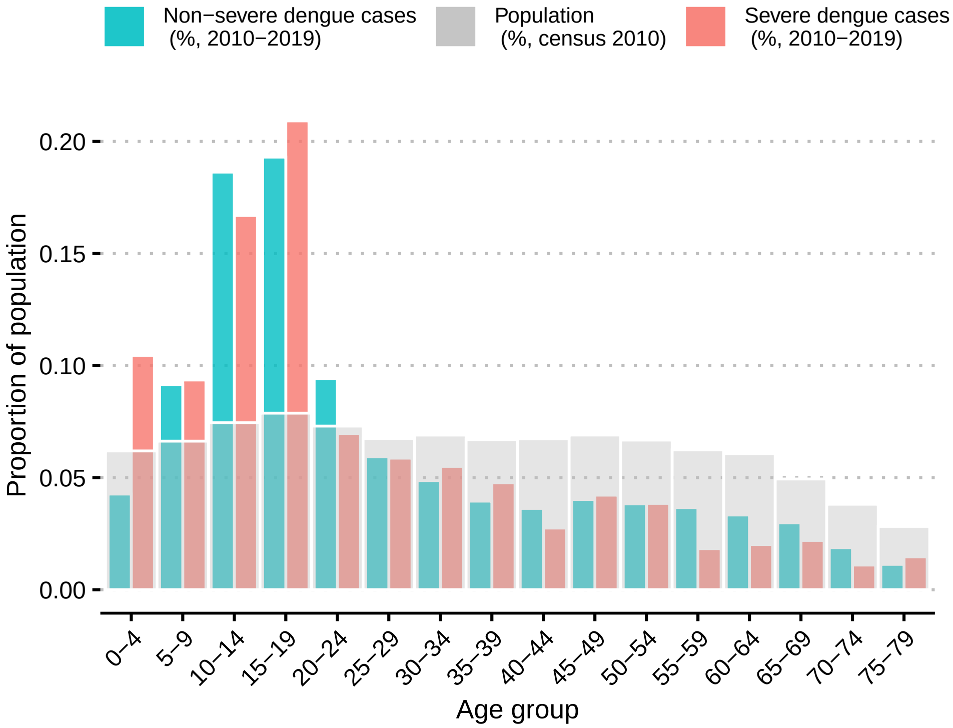

Fig B. Age distribution of reported non-severe and severe dengue cases (colored bars) in Puerto Rico from 2010 to 2019. The grey bars represent the age distribution of the population (US census, 2010).

A

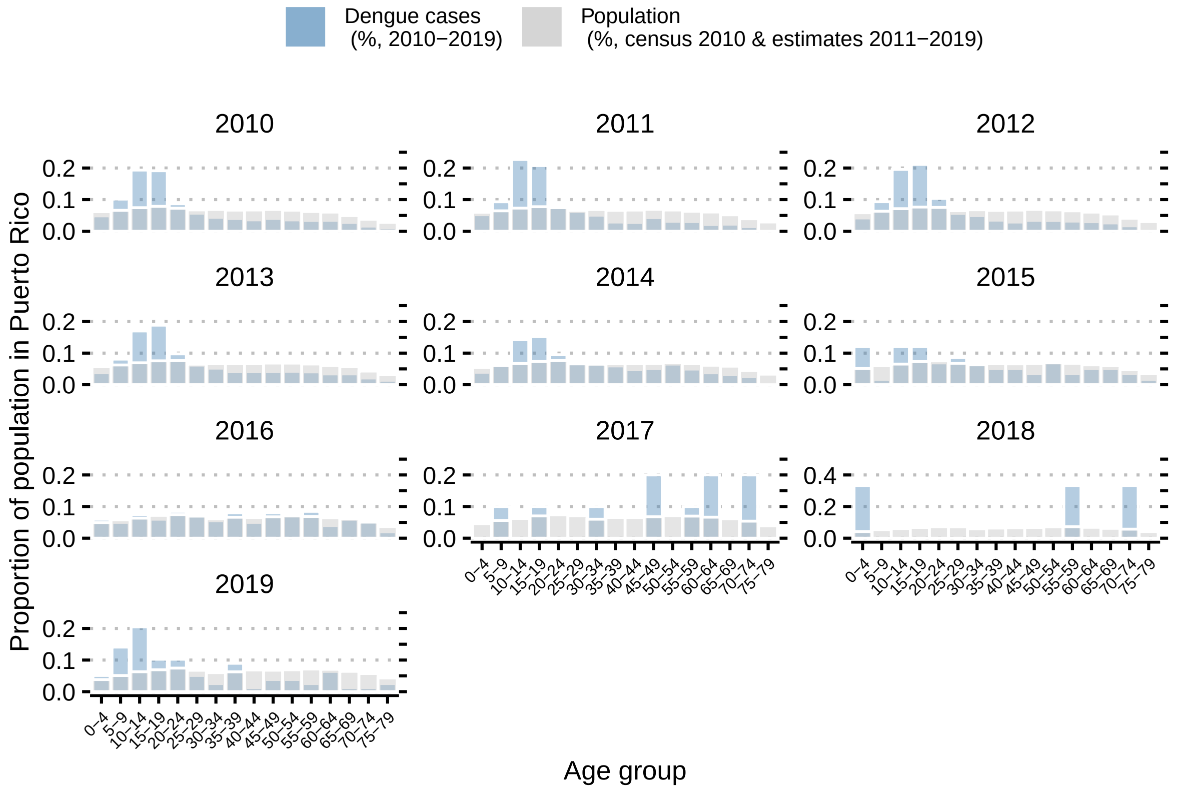

Dengue cases
(%, 2010−2019)
Population
(%, census 2010 & estimates 2011−2019)
2010
2011
2012
2013
2014
2015
2016
2017
2018
2019
Proportion of population in Puerto Rico
Age group

B

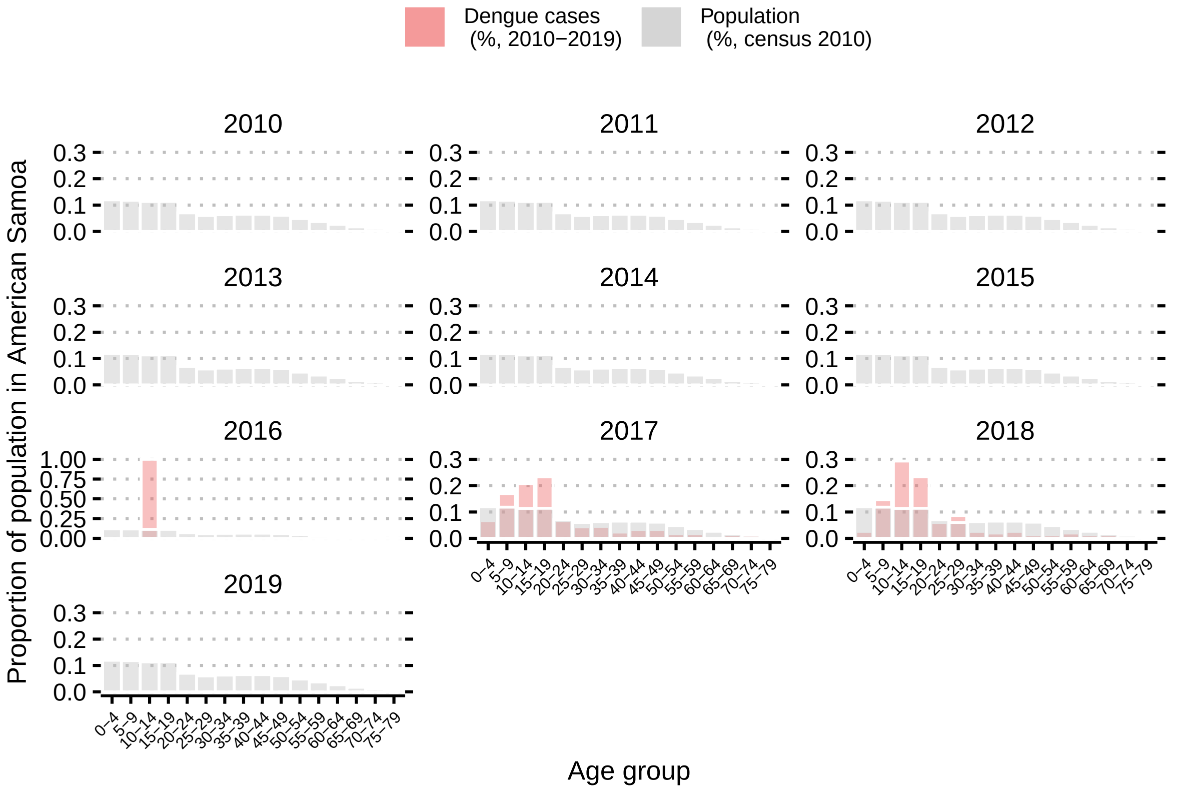

Dengue cases
(%, 2010−2019)
Population
(%, census 2010)
2010
2011
2012
2013
2014
2015
2016
2017
2018
2019
Proportion of population in American Samoa
Age group

C

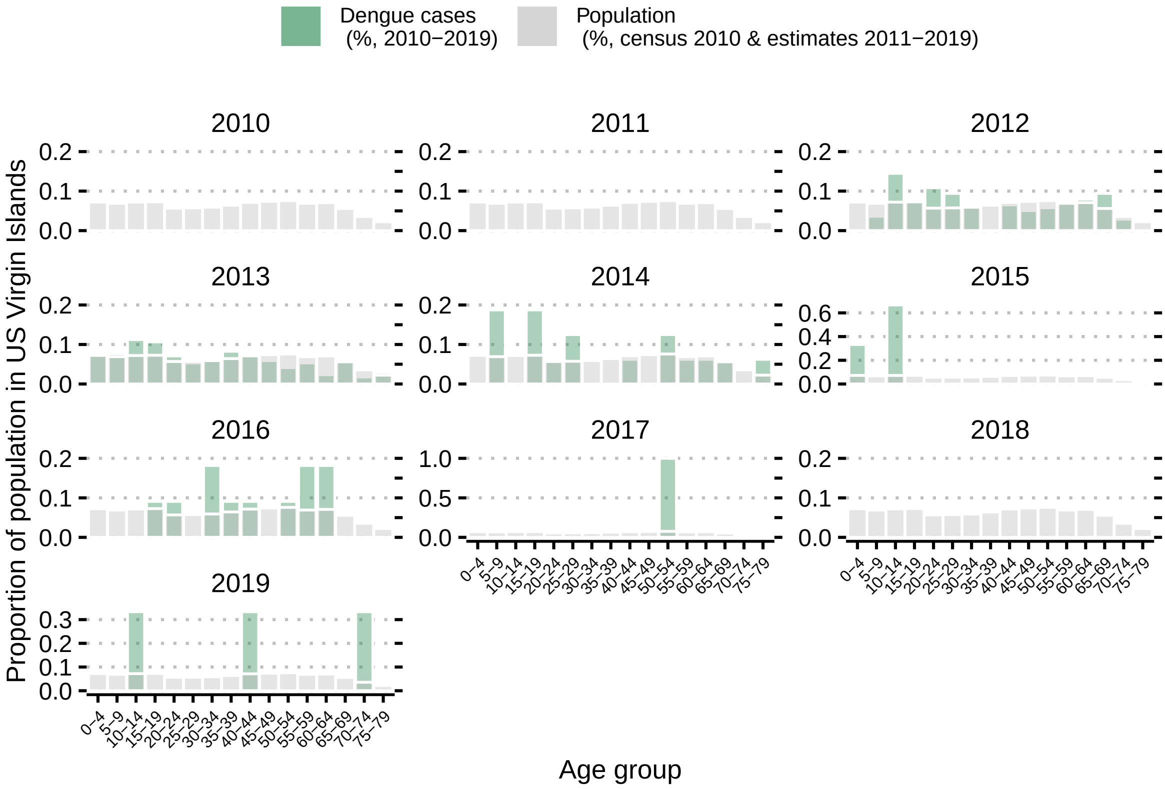
Dengue cases
(%, 2010−2019)
Population
(%, census 2010 & estimates 2011−2019)
2010
2011
2012
2013
2014
2015
2016
2017
2018
2019
Proportion of population in US Virgin Islands
Age group
0−4
5−9
10−14
15−19
20−24
25−29
30−34
35−39
40−44
45−49
50−54
55−59
60−64
65−69
70−74
75−79

D

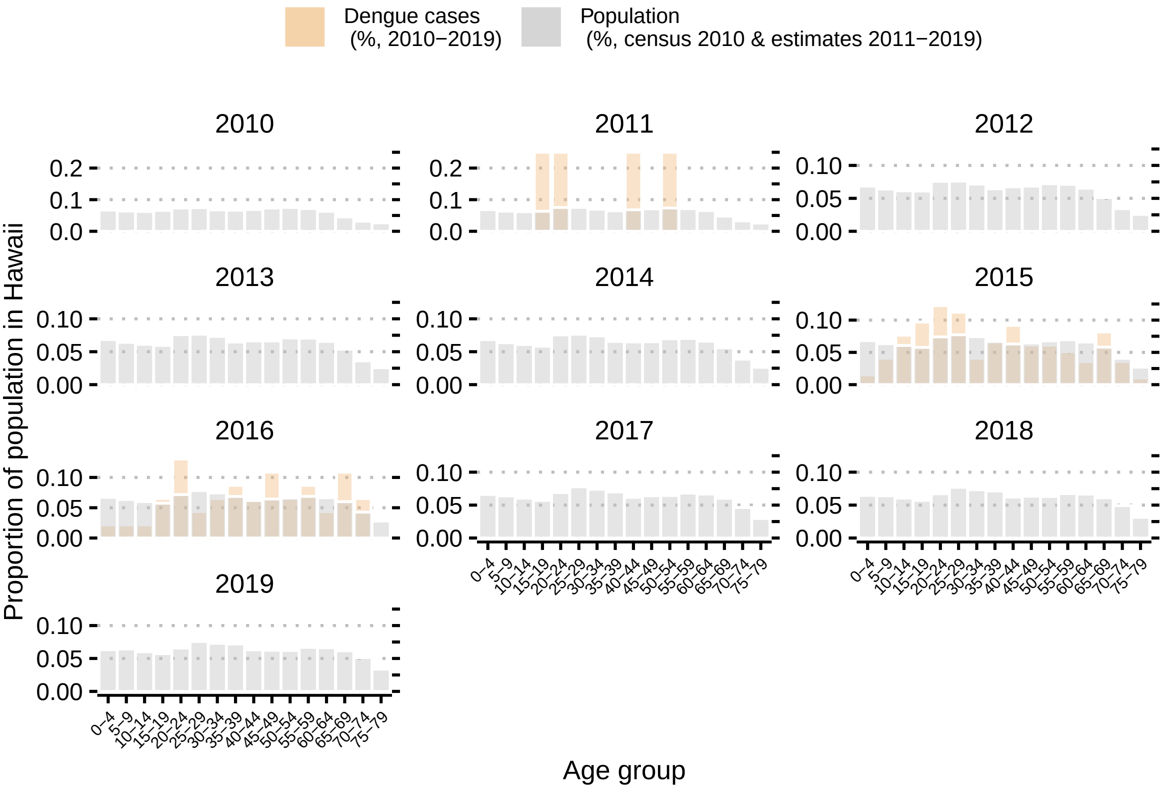
Dengue cases
(%, 2010−2019)
Population
(%, census 2010 & estimates 2011−2019)
2010
2011
2012
2013
2014
2015
2016
2017
2018
2019
Proportion of population in Hawaii
Age group
0−4
5−9
10−14
15−19
20−24
25−29
30−34
35−39
40−44
45−49
50−54
55−59
60−64
65−69
70−74
75−79

E

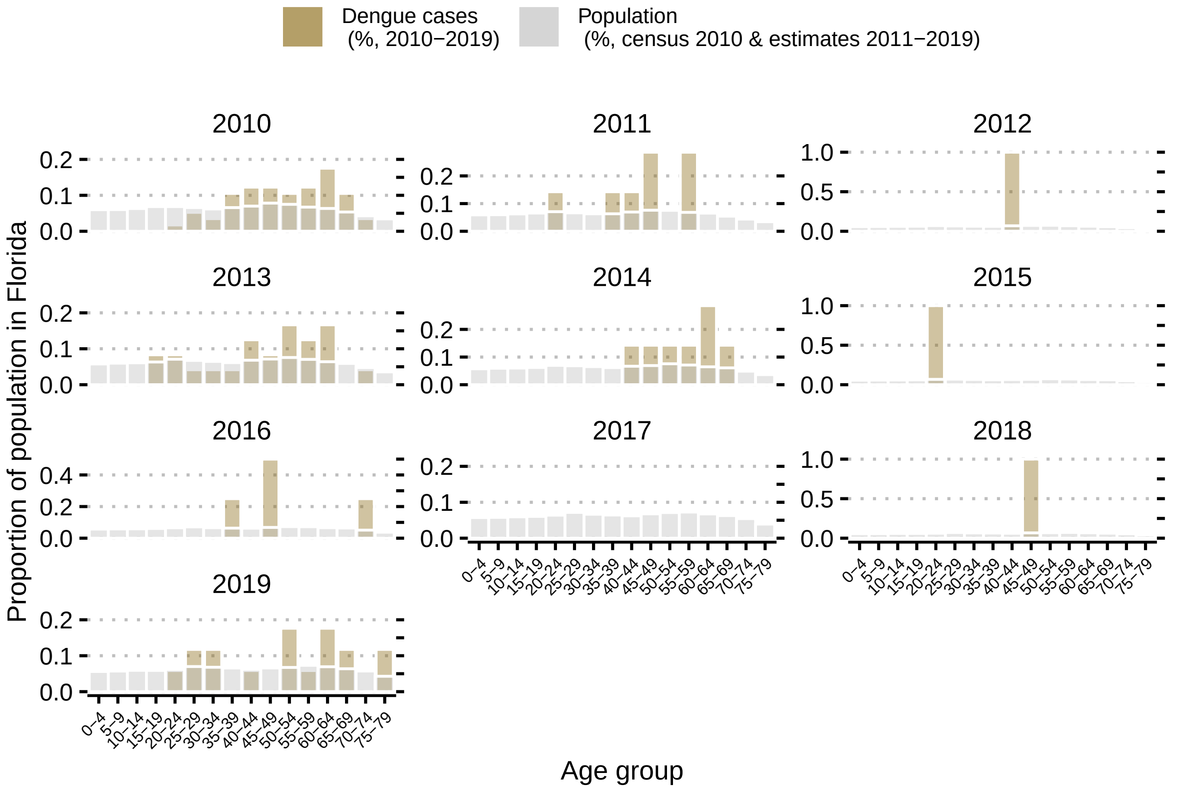

F

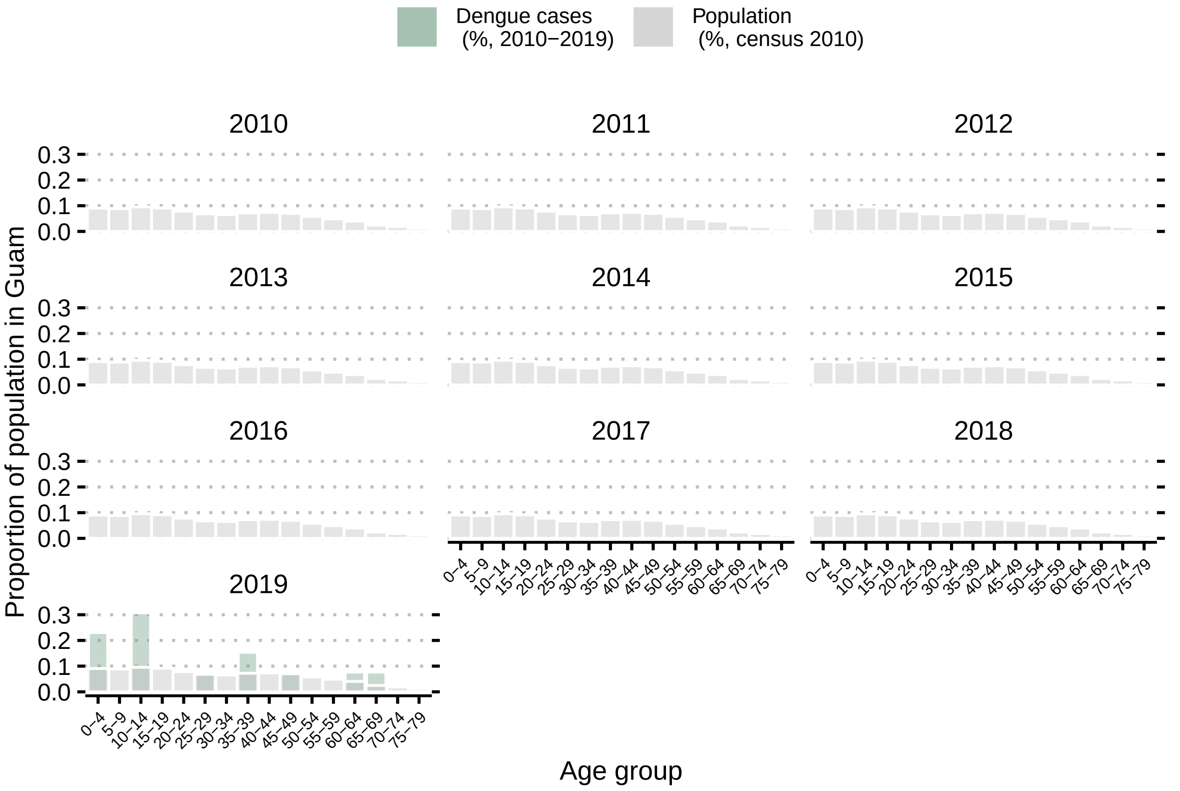

Fig C. Yearly age distribution of dengue reported cases in Puerto Rico (A), American Samoa

(B), US Virgin Islands (C), Hawaii (D), Florida (E) and Guam (F). Scales may be adjusted for years with fewer dengue cases reported.

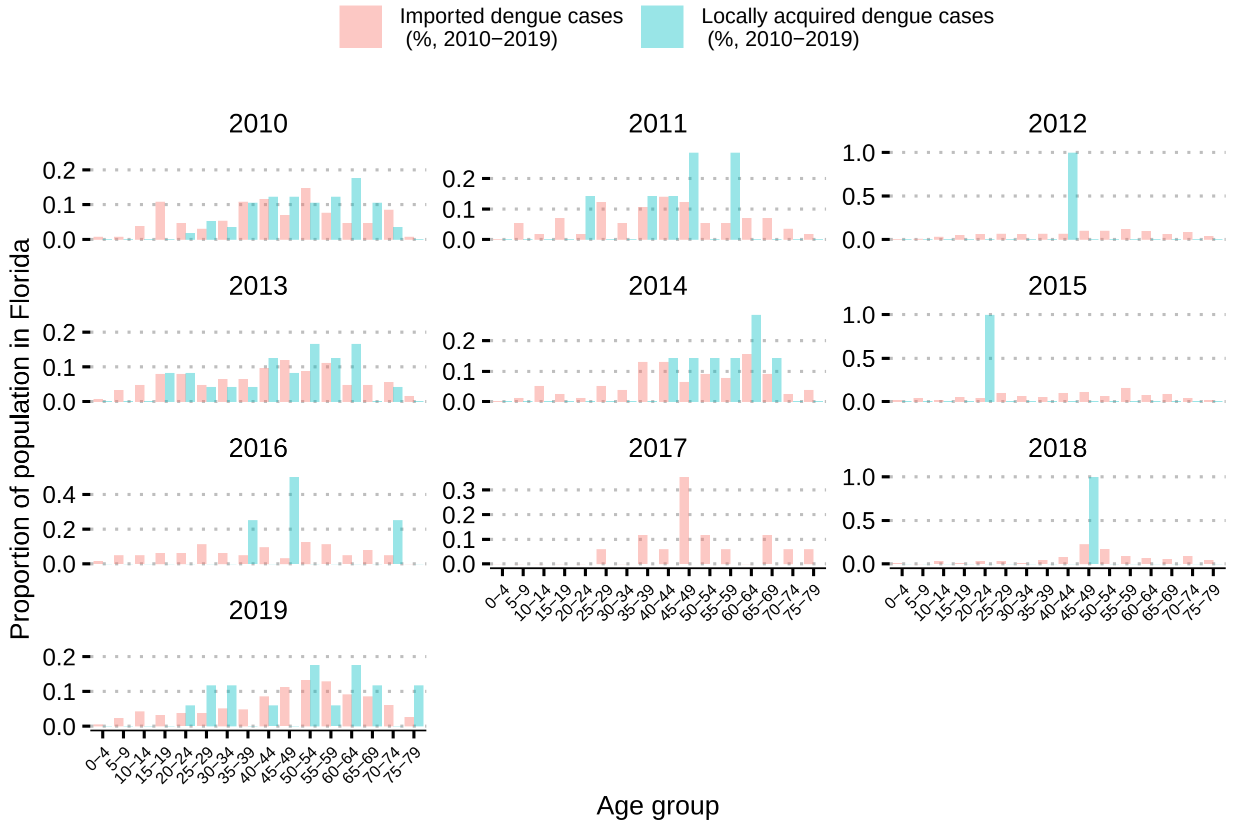

Fig D. Yearly age distribution of locally-acquired and imported dengue reported cases in Florida. Scales are adjusted for years with fewer dengue cases reported.

2.2 Parameter identifiability

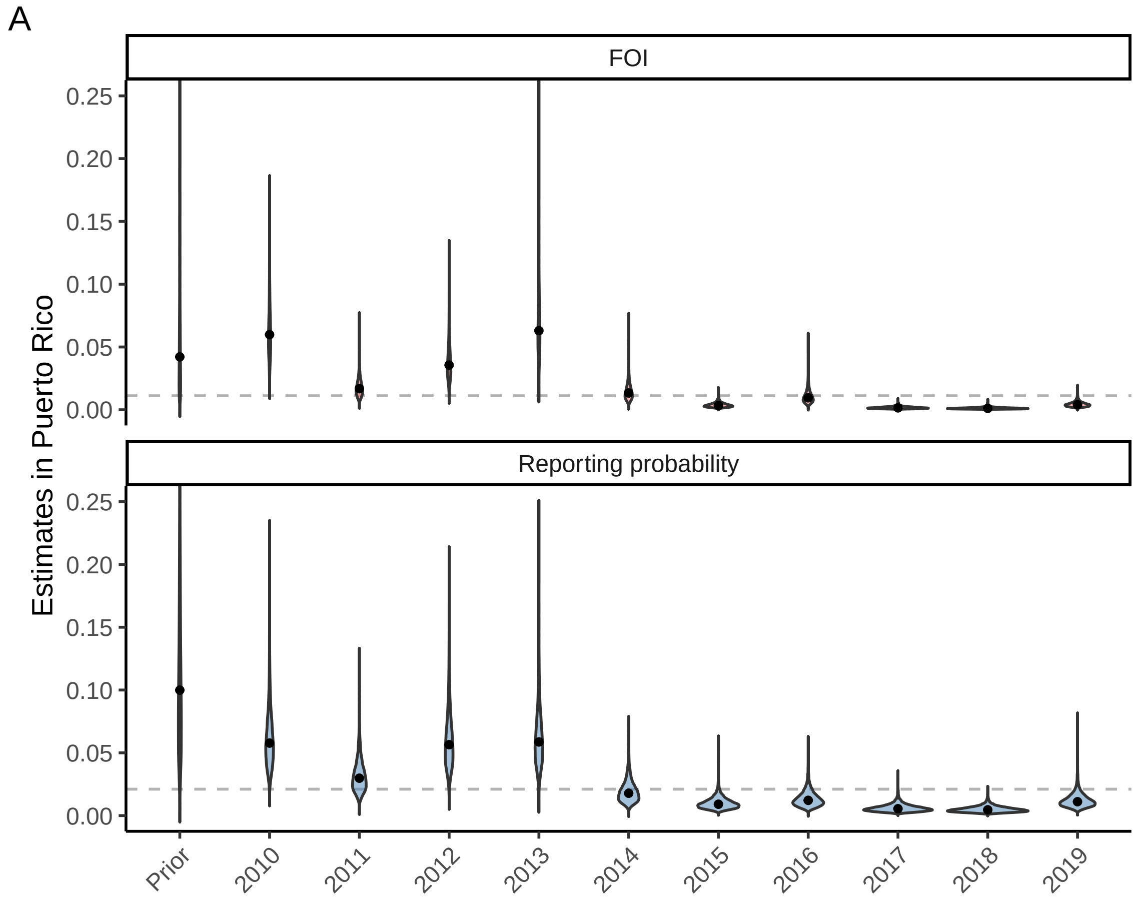

B

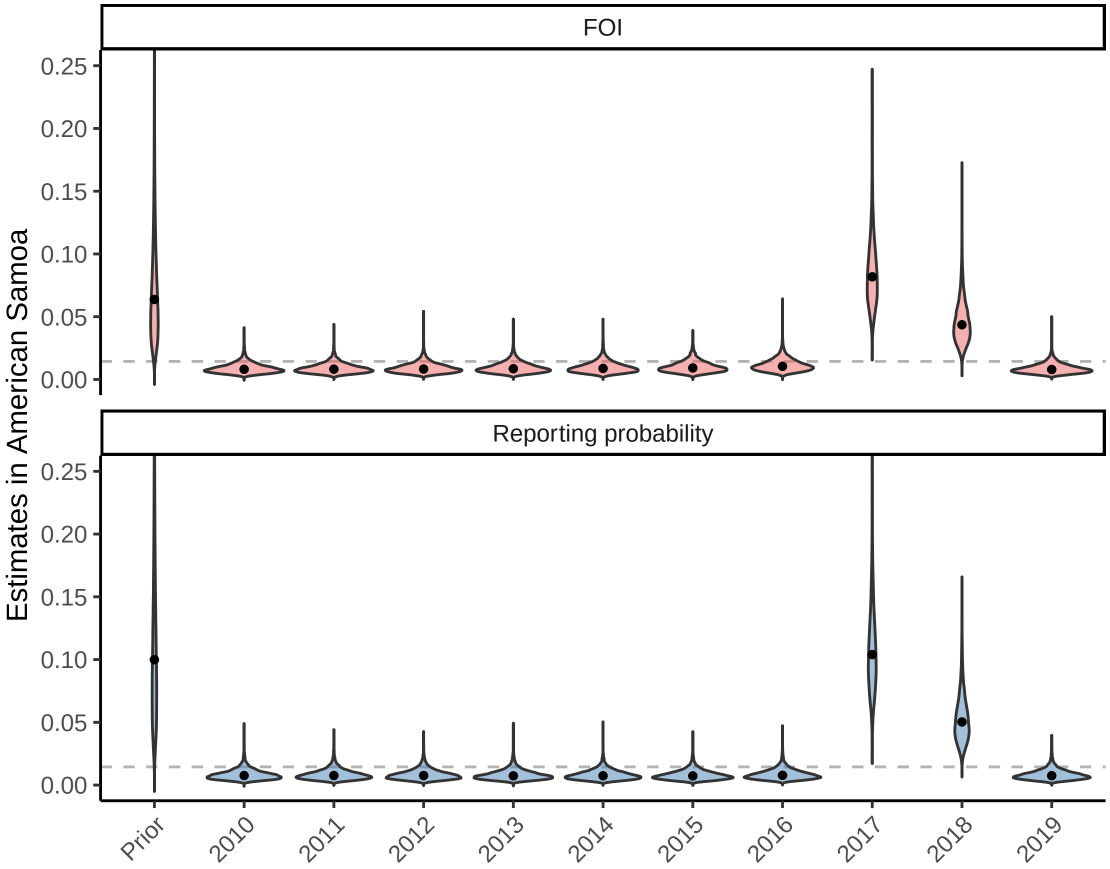
FOI
Reporting probability
Estimates in American Samoa
0.25
0.20
0.15
0.10
0.05
0.00
Prior
2010
2011
2012
2013
2014
2015
2016
2017
2018
2019

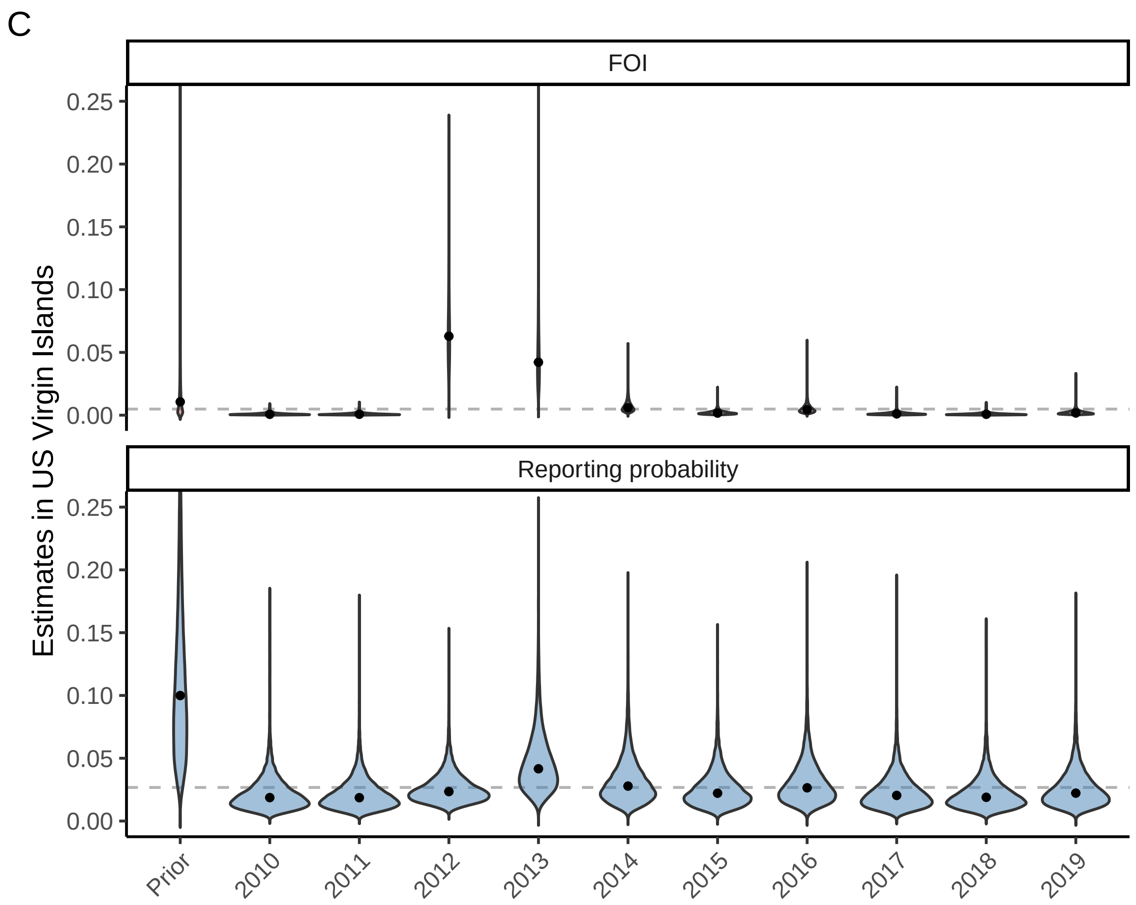

C
FOI
Reporting probability
Estimates in US Virgin Islands
0.25
0.20
0.15
0.10
0.05
0.00
Prior
2010
2011
2012
2013
2014
2015
2016
2017
2018
2019

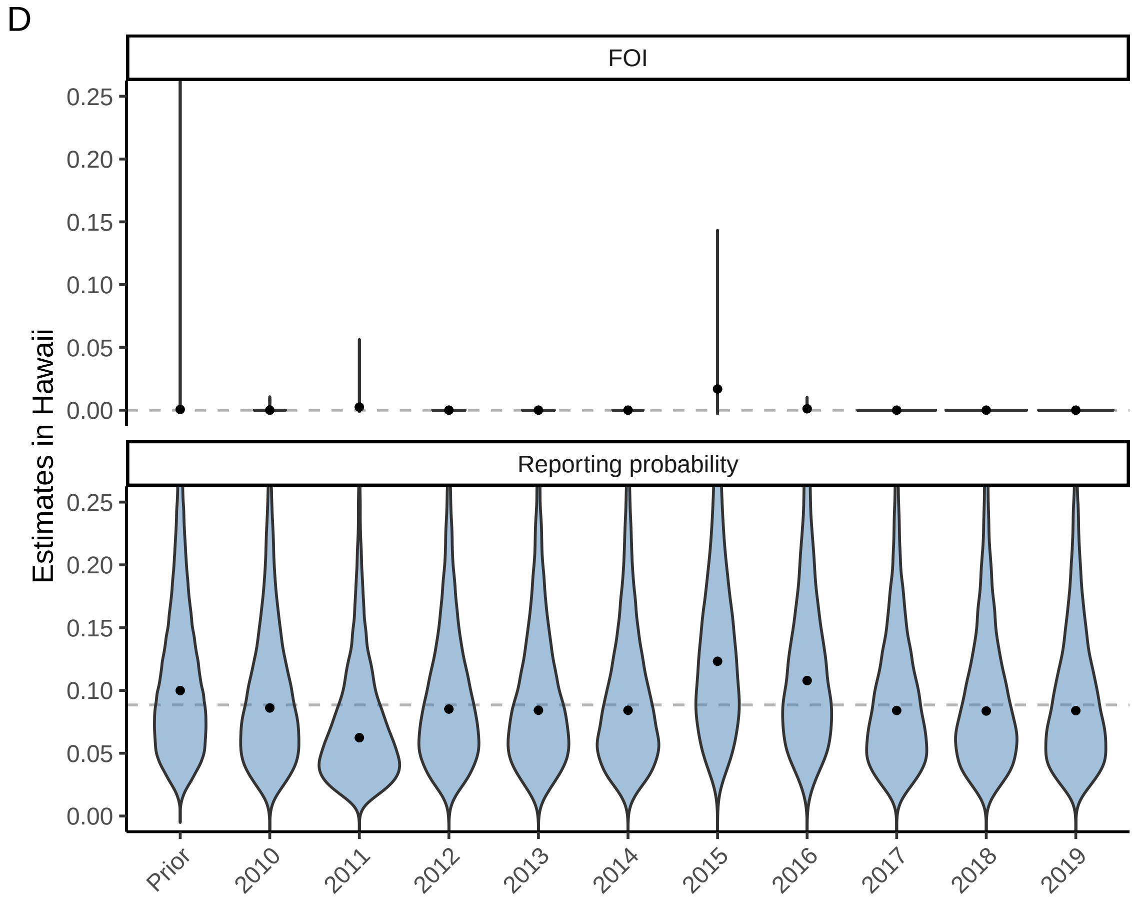
D
FOI
Reporting probability
Estimates in Hawaii
0.25
0.20
0.15
0.10
0.05
0.00
Prior
2010
2011
2012
2013
2014
2015
2016
2017
2018
2019

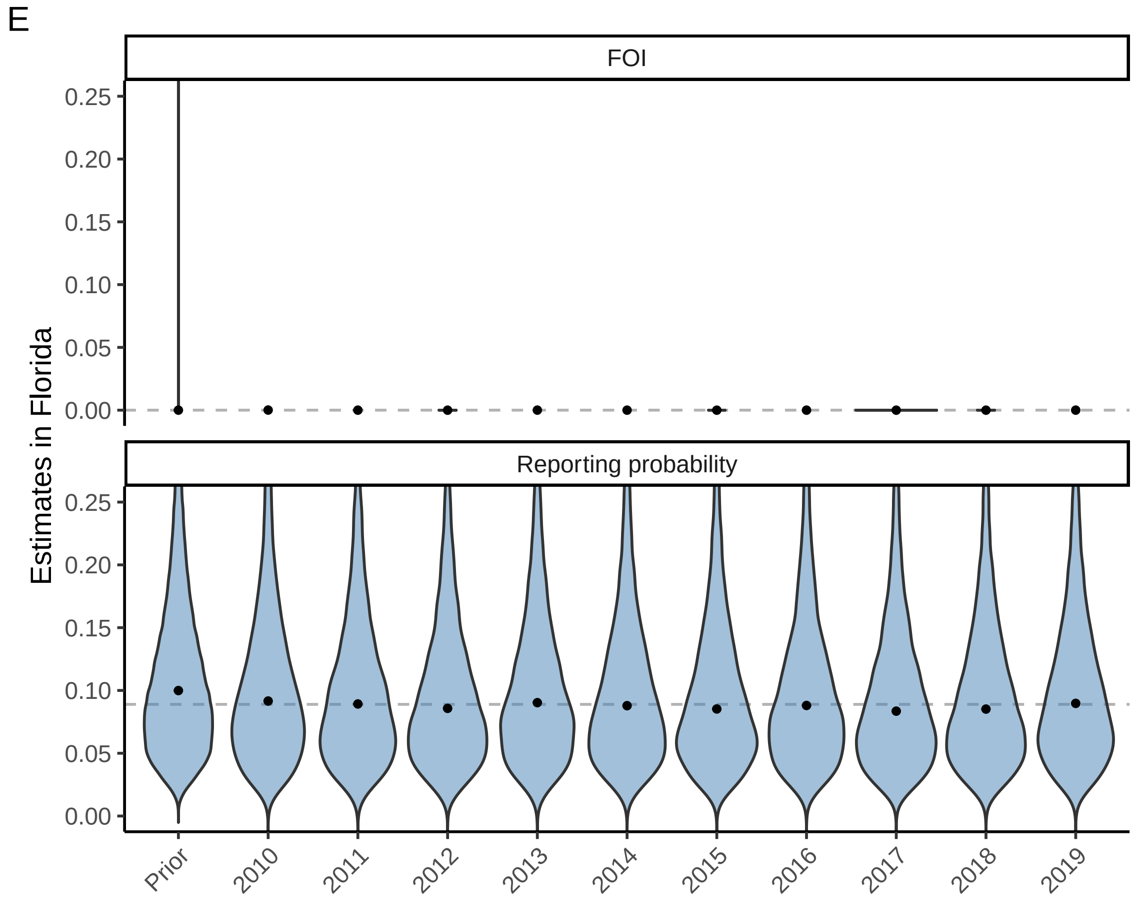
E
FOI
Reporting probability
Estimates in Florida
0.25
0.20
0.15
0.10
0.05
0.00
Prior
2010
2011
2012
2013
2014
2015
2016
2017
2018
2019

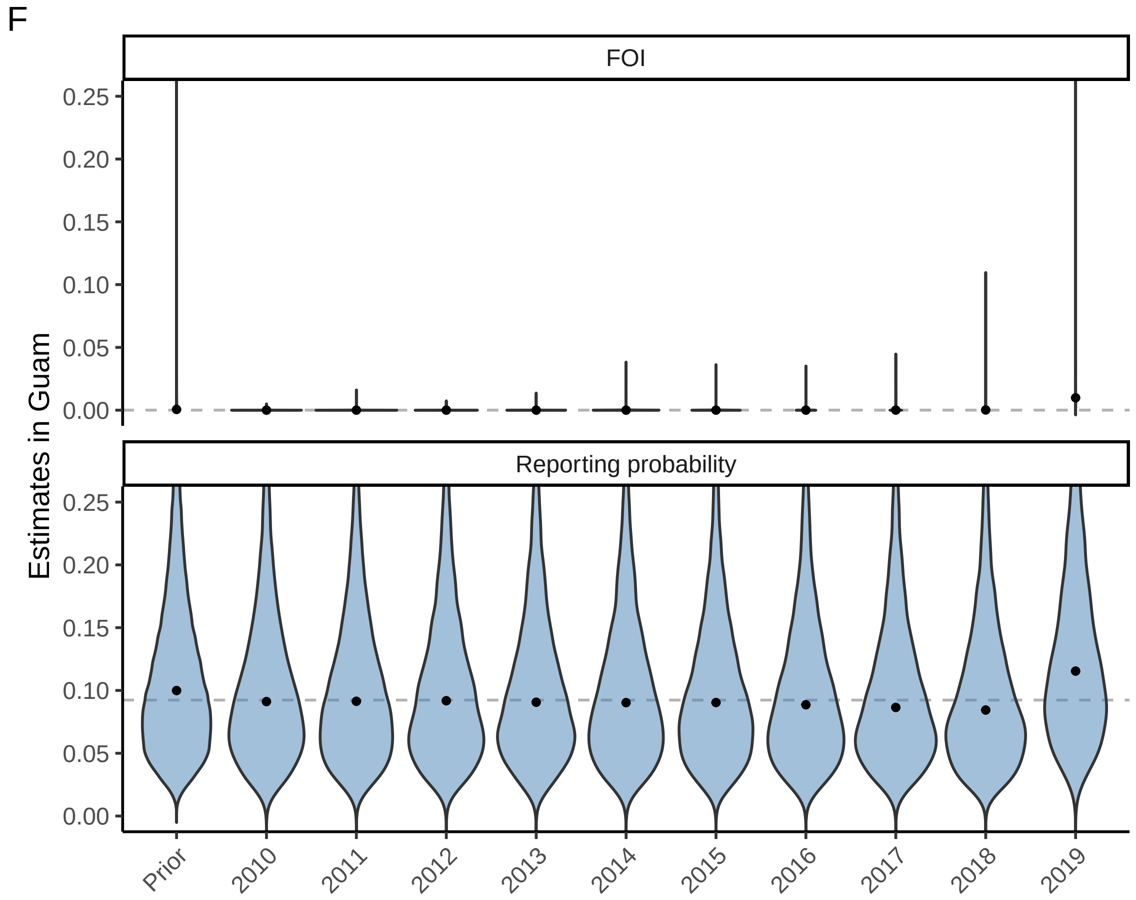

Fig E. Violin plots of FOI and reporting probability prior and posterior estimates in Puerto Rico (A), American Samoa (B), the US Virgin Islands (C), Hawaii (D), Florida (E) and Guam (F) from 2010 to 2019. Points represent the median value of each estimate and the dashed lines represent the long term average of FOI and reporting probability estimates.

2.3 Model comparison using log likelihood

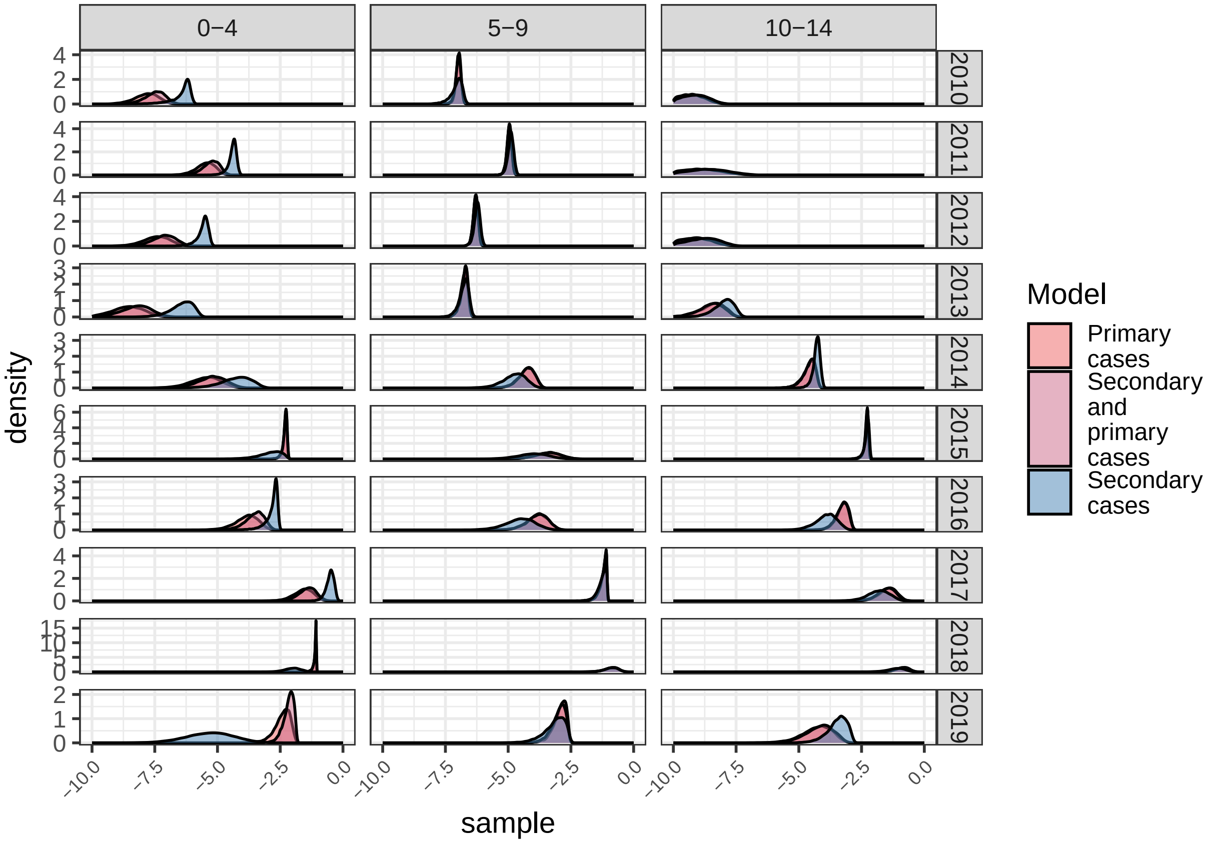

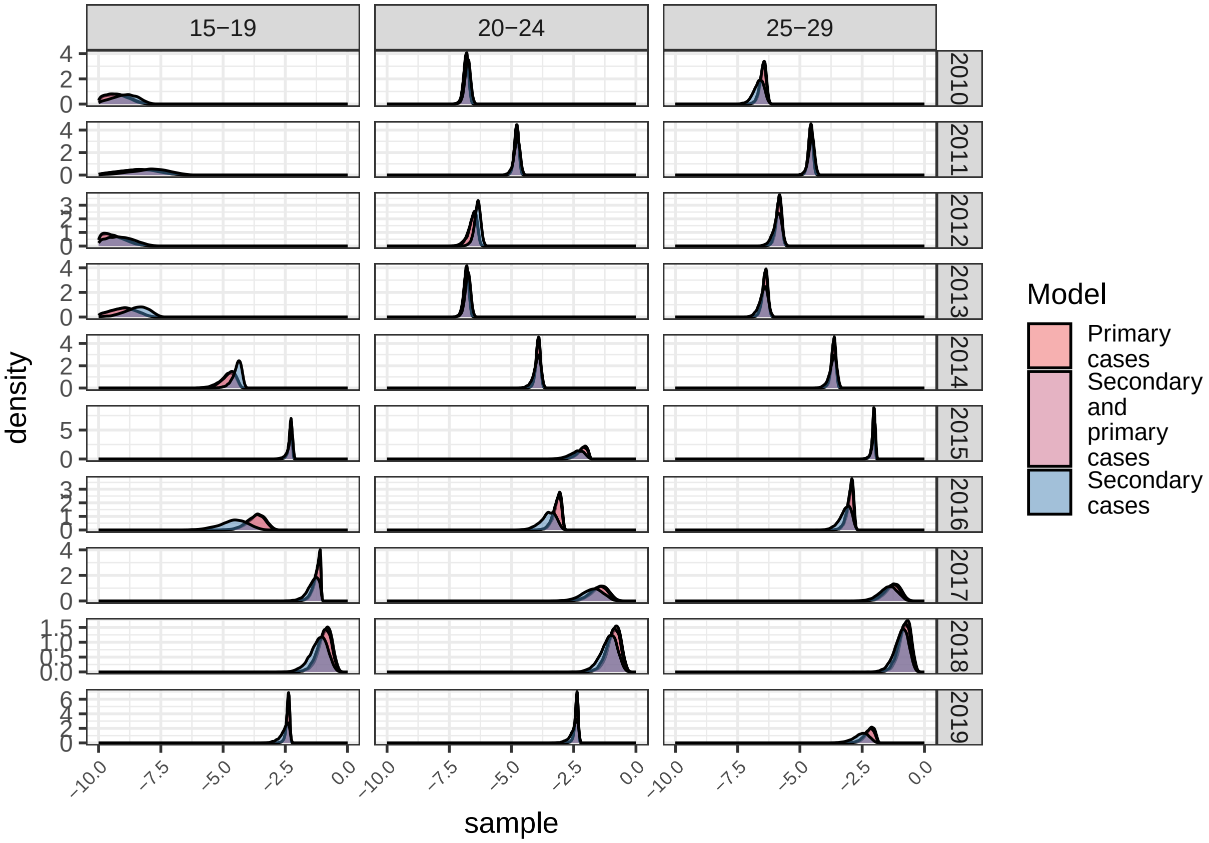

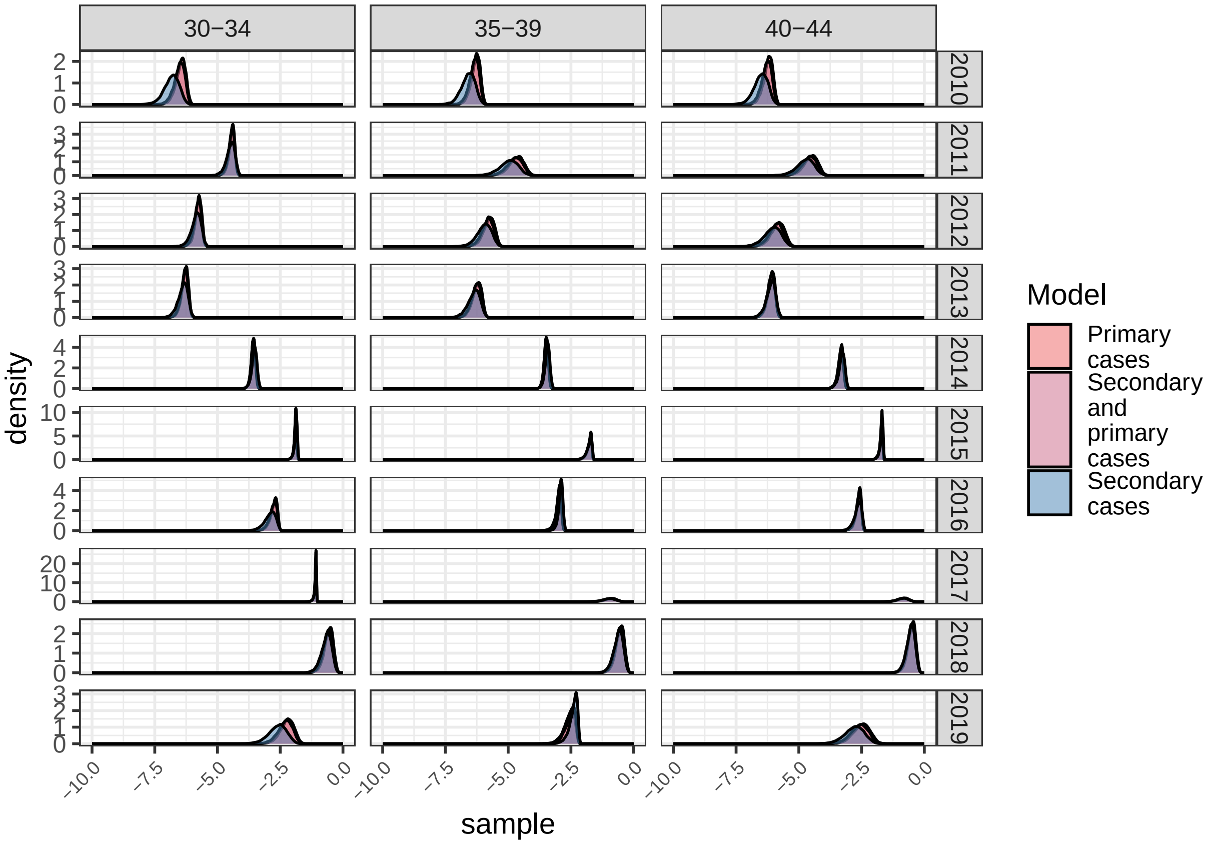
30−34
35−39
40−44
2010
2011
2012
2013
2014
2015
2016
2017
2018
2019
density
sample
−10.0
−7.5
−5.0
−2.5
0.0
Model
Primary cases
Secondary and primary cases
Secondary cases

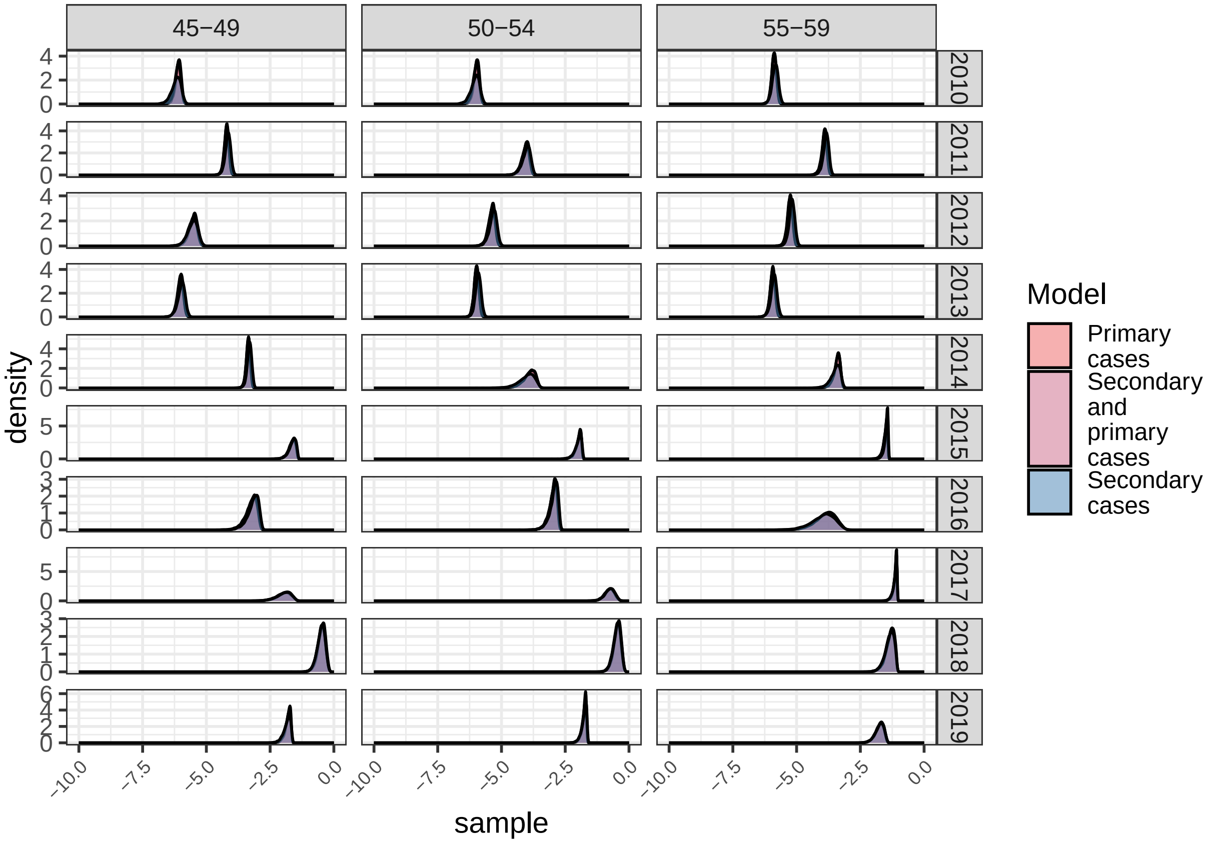
45−49
50−54
55−59
2010
2011
2012
2013
2014
2015
2016
2017
2018
2019
density
sample
−10.0
−7.5
−5.0
−2.5
0.0
Model
Primary cases
Secondary and primary cases
Secondary cases

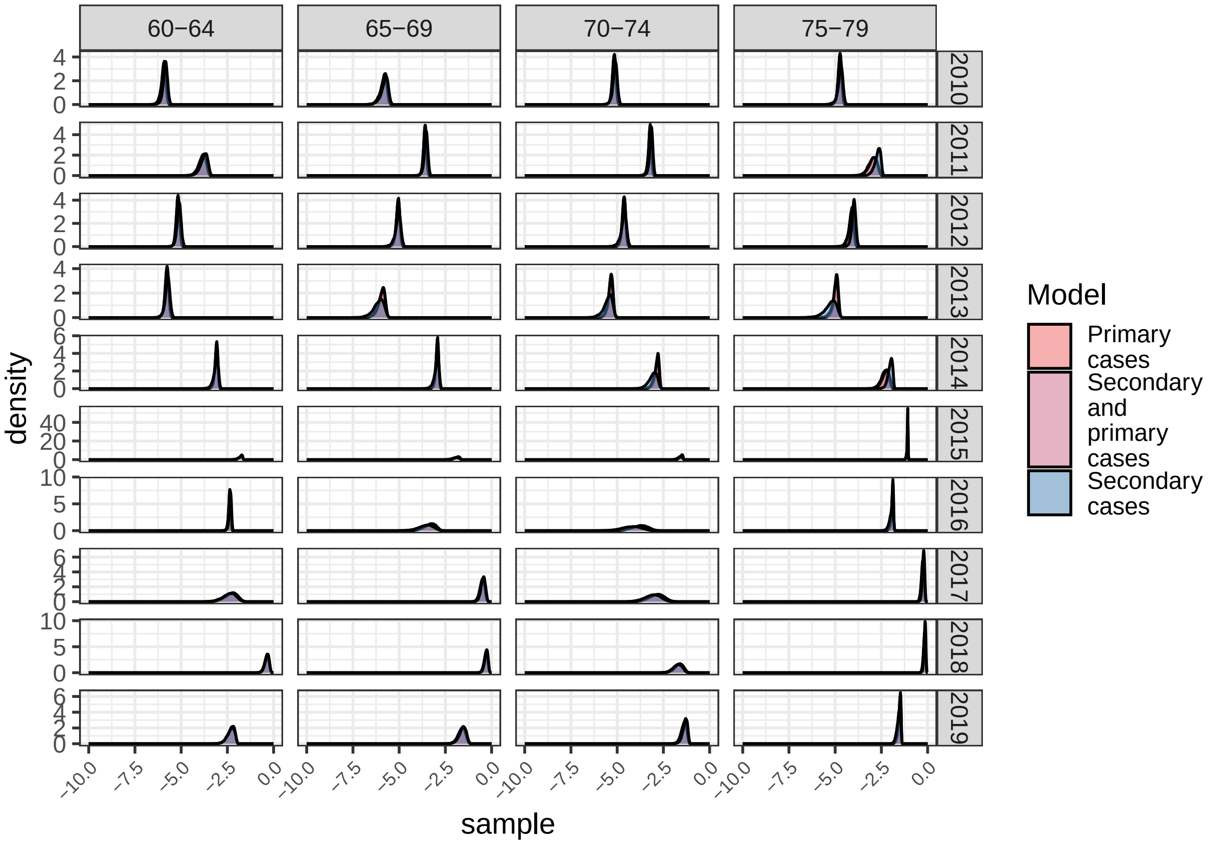

Fig F. Comparison of log likelihood samples by age group and years of models Primary, Primary & Secondary, and Secondary using Puerto Rico data. Models's log-likelihood values may overlap. The log likelihood value in a model is a measure of goodness of fit. The higher the value (i.e., closer to 0), the better.

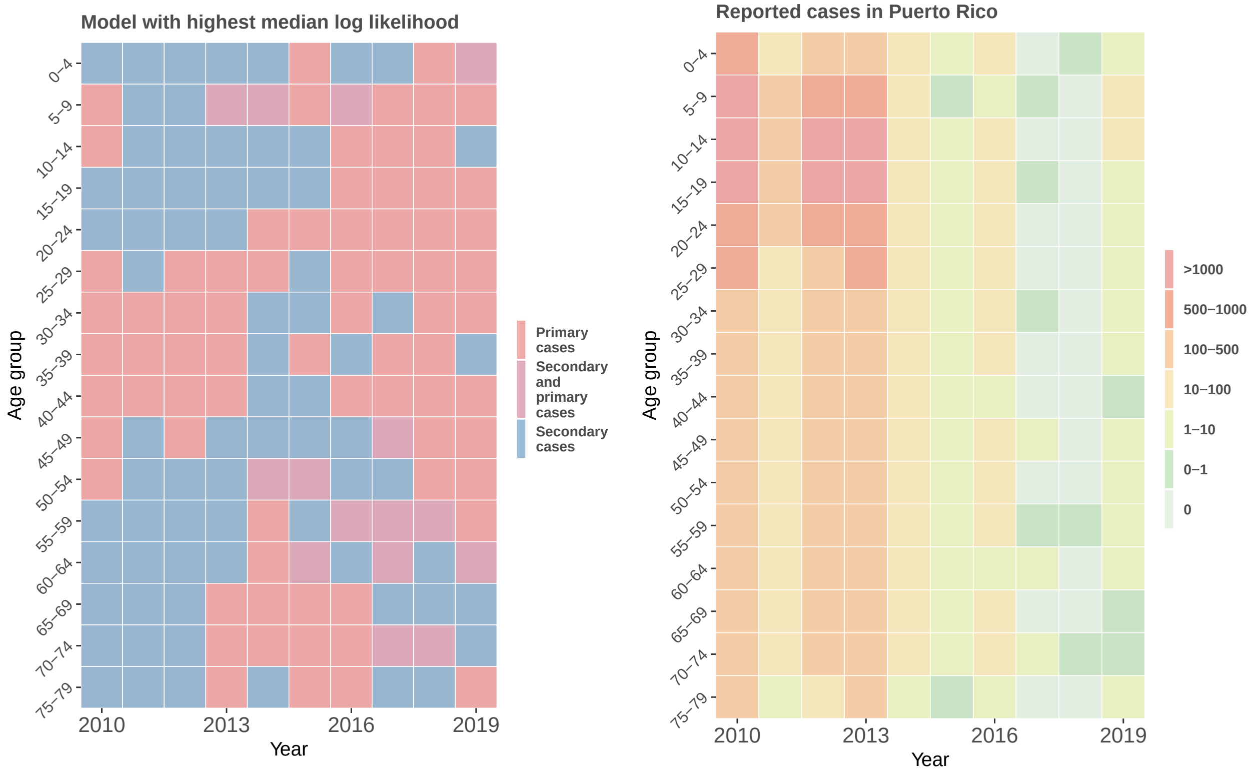

Fig G. Heatmap comparison between models Primary, Primary & Secondary, and Secondary of log likelihood median samples and cases by age group and years using Puerto Rico data. The log likelihood value in a model is a measure of goodness of fit. Here, models with log likelihood values closest to 0 were plotted.

2.4 Fit to reported cases

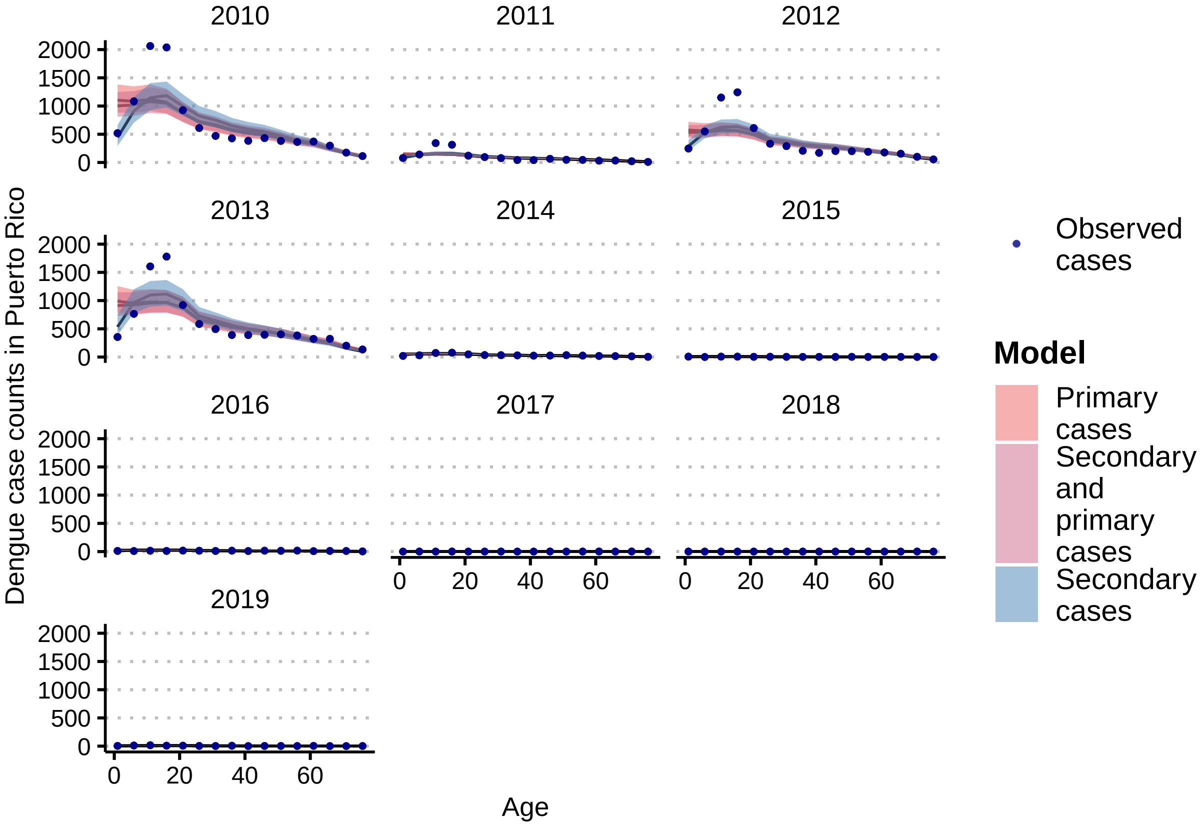

Fig H. Reported cases model fit (all dengue cases) by age group in Puerto Rico from 2010 to 2019. Points represent cases reported to ArboNET while lines represent model fit to Primary, Primary & Secondary, and Secondary models. Shaded area represent the best model 95% CrI.

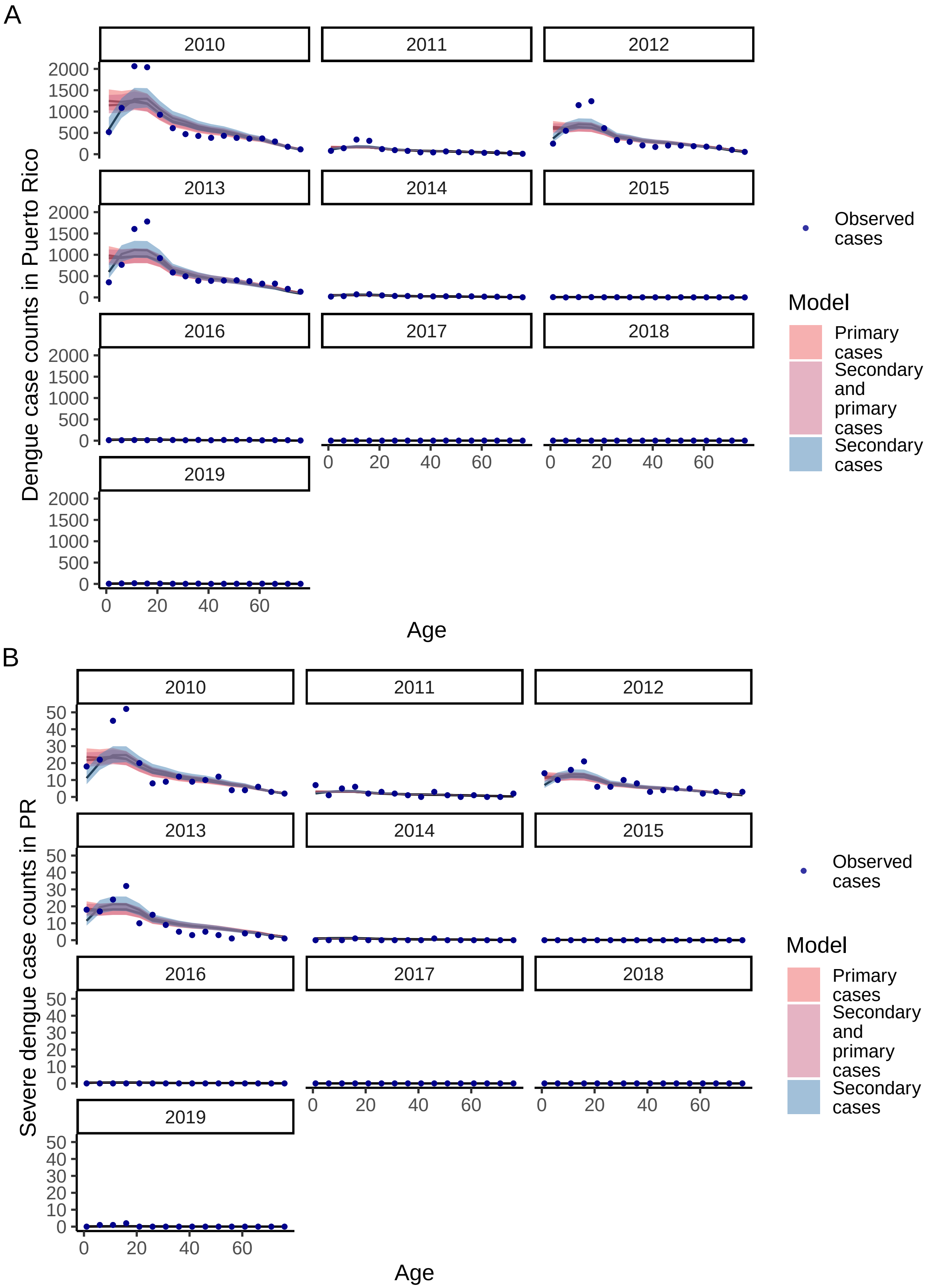

Fig I. Reported cases model fit to model fitted to all dengue (A) and severe cases (B) by age

group in Puerto Rico from 2010 to 2019. Points represent dengue (A) and severe cases (B) reported to ArboNET while lines represent model fit to Primary, Primary & Secondary, and Secondary models. Shaded areas represent the best model 95% CrI.

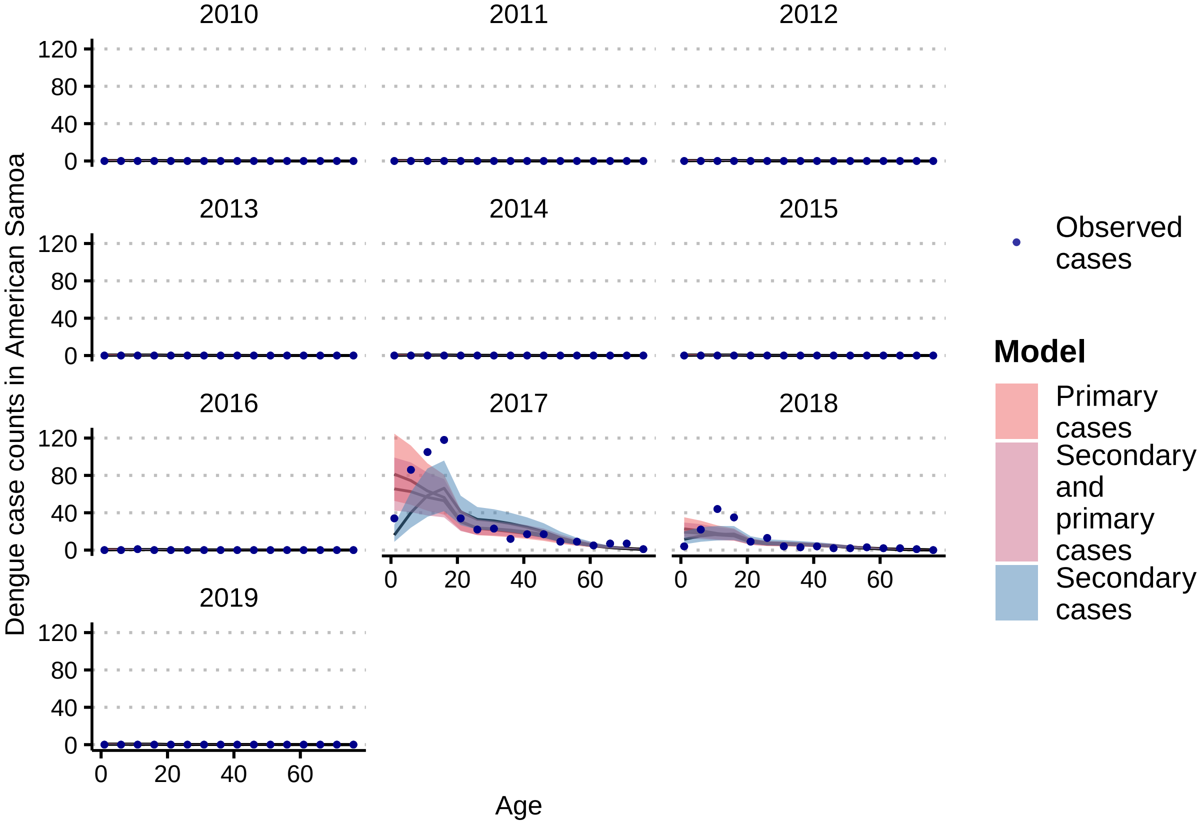

Fig J. Reported cases model fit by age group in American Samoa from 2010 to 2019. Points represent cases reported to ArboNET while lines represent model fit to Primary, Primary & Secondary, and Secondary models. Shaded area represent the best model 95% CrI.

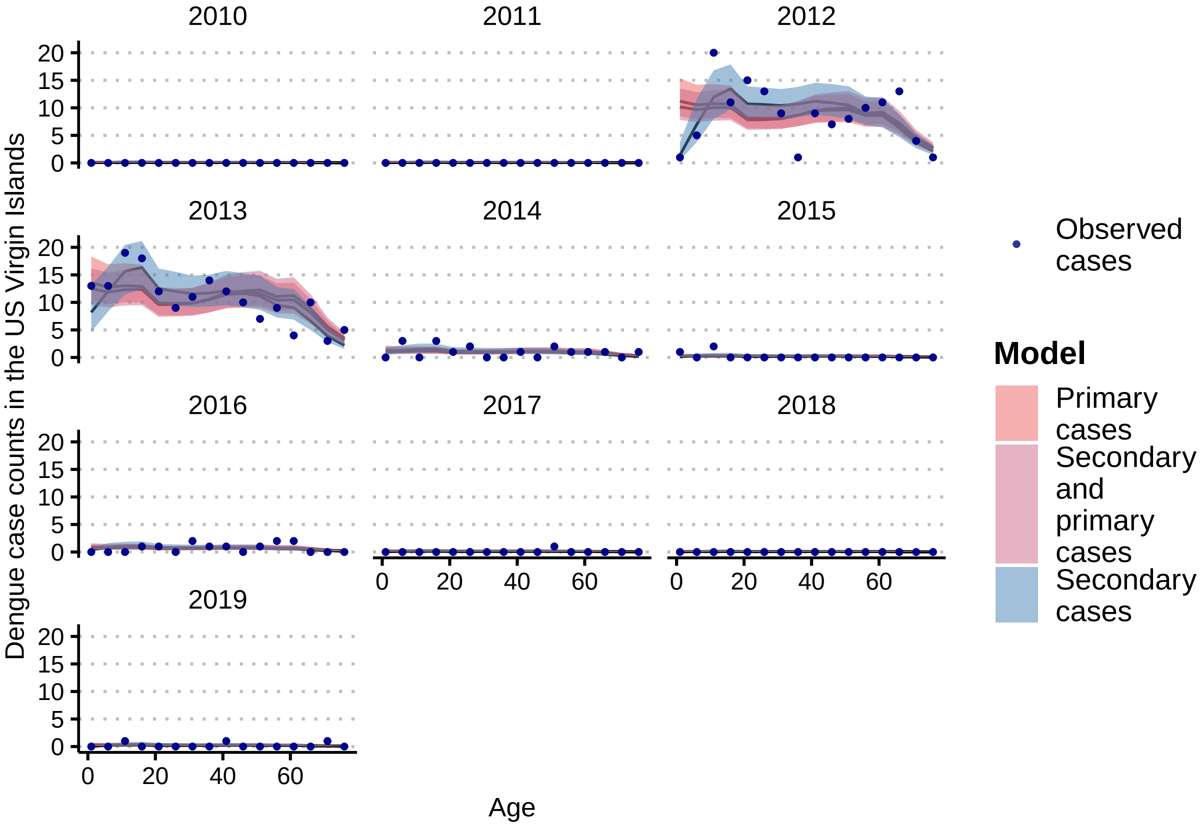

Fig K. Reported cases model fit by age group in US Virgin Islands from 2010 to 2019. Points represent cases reported to ArboNET while lines represent model fit to Primary, Primary & Secondary, and Secondary models. Shaded area represent the best model 95% CrI.

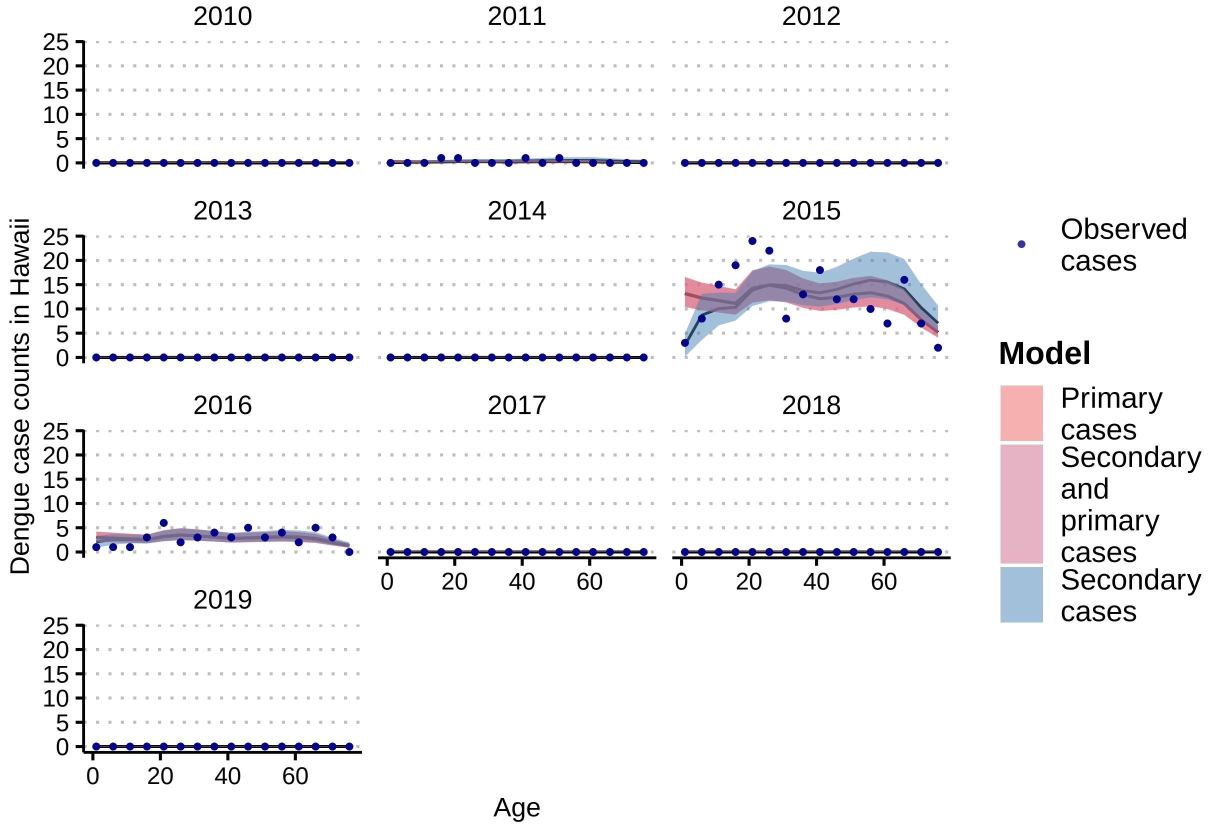

Fig L. Reported cases model fit by age group in Hawaii from 2010 to 2019. Points represent cases reported to ArboNET while lines represent model fit to Primary, Primary & Secondary, and Secondary models. Shaded area represent the best model 95% CrI.

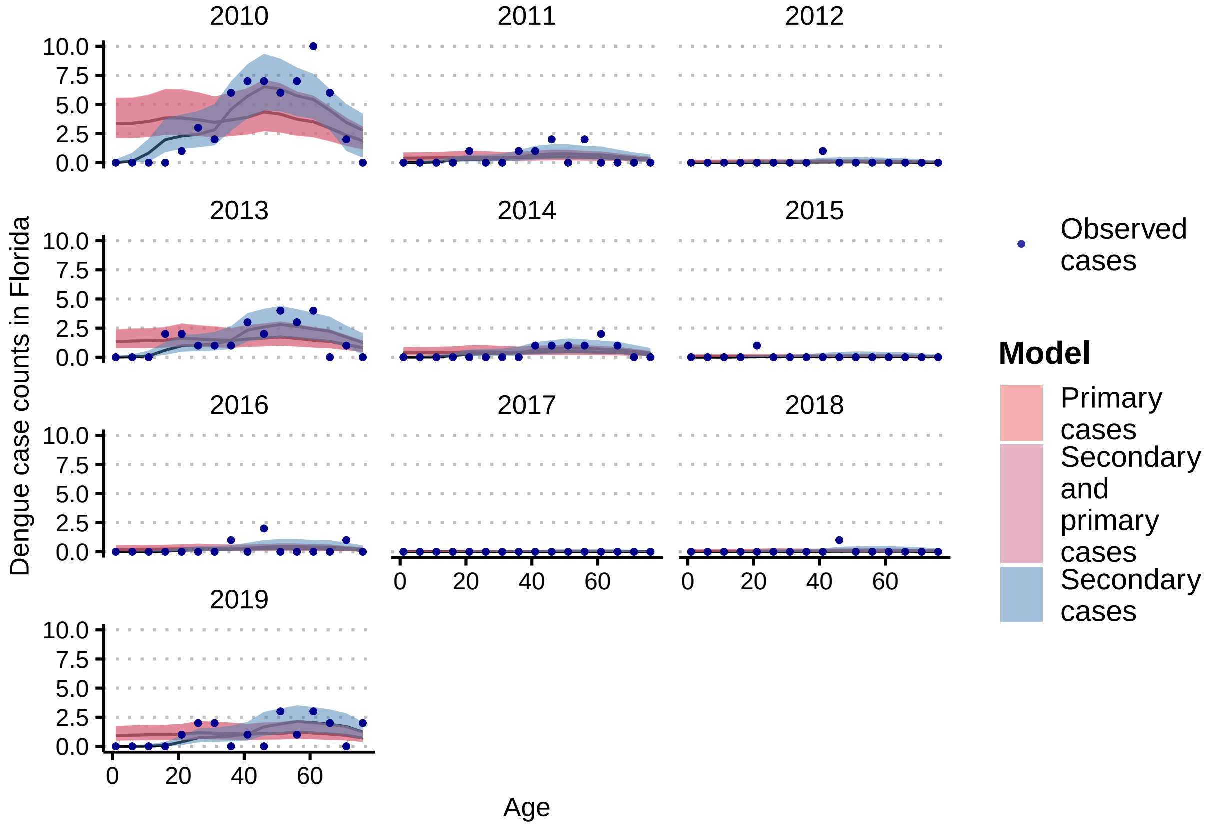

Fig M. Reported cases model fit by age group in Florida from 2010 to 2019. Points represent cases reported to ArboNET while lines represent model fit to Primary, Primary & Secondary, and Secondary models. Shaded area represent the best model 95% CrI.

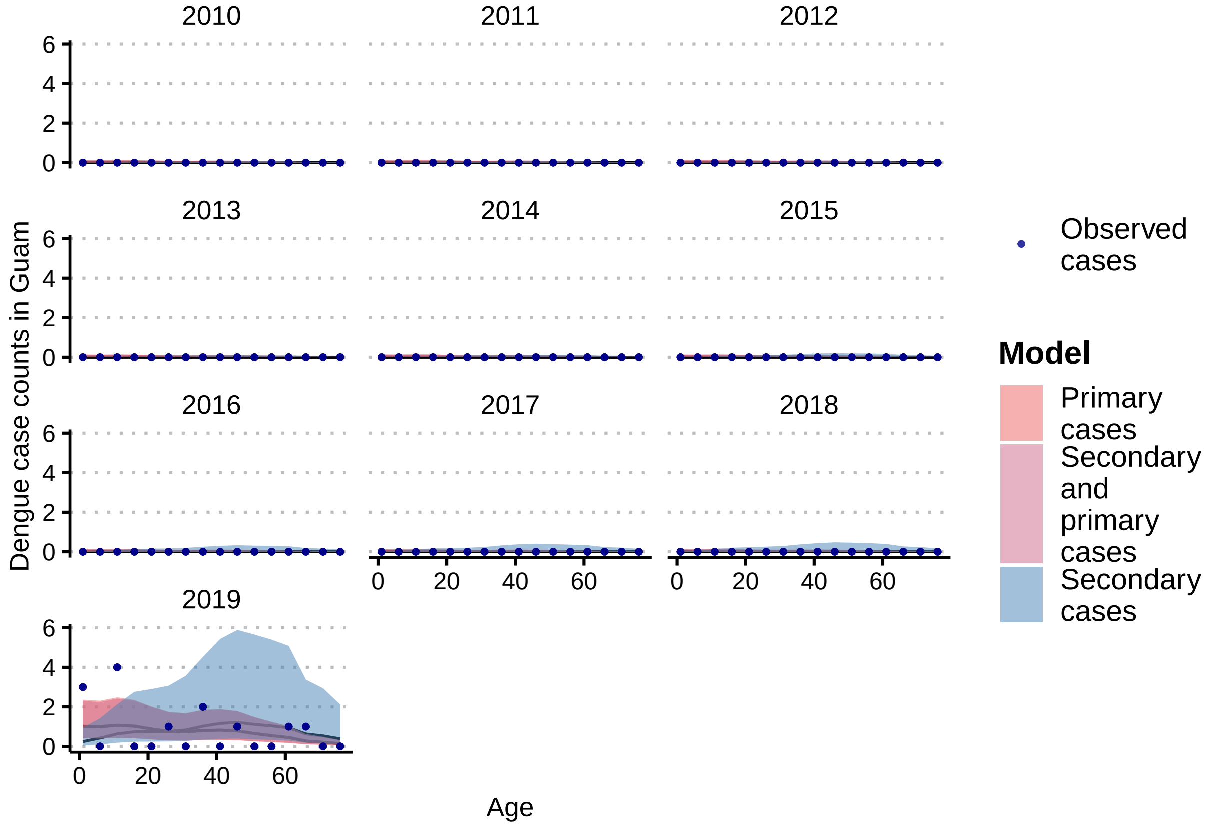

Fig N. Reported cases model fit by age group in Guam from 2010 to 2019. Points represent cases reported to ArboNET while lines represent model fit to Primary, Primary & Secondary, and Secondary models. Shaded areas represent the best model 95% CrI.

2.5 Other parameters

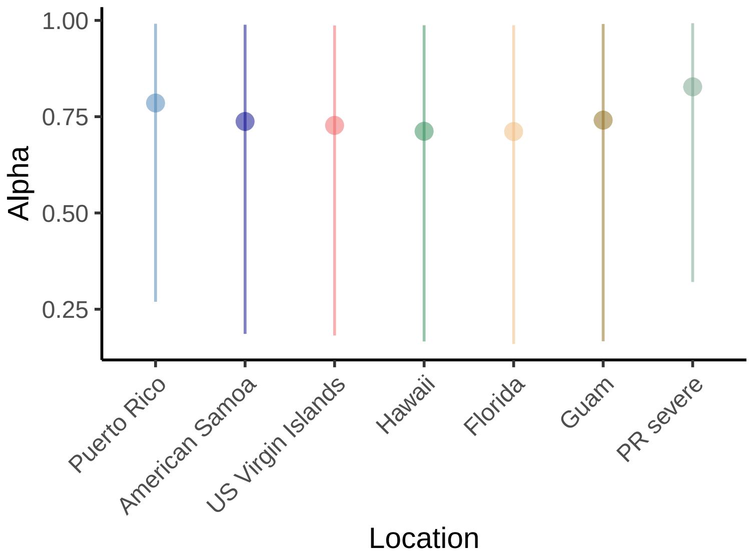

Fig O. Alpha parameter posterior estimates with 95% CrI (vertical bars) in Puerto Rico, Puerto Rico severe (using severe cases), American Samoa, US Virgin Islands, Hawaii, Florida and Guam.

Table B. Force of infection estimates in Puerto Rico, American Samoa, US Virgin Islands, Hawaii, Florida and Guam from 2010 to 2019. Median and 95 % Bayesian credible intervals.

Location	Year	Force of Infection (95% CrI)
**PR**	2010	0.05983 (0.03109-0.11273)
	2011	0.01677 (0.00792-0.03433)
	2012	0.0356 (0.01815-0.06911)
	2013	0.06303 (0.03246-0.12146)
	2014	0.01333 (0.0058-0.03112)
	2015	0.00339 (0.00147-0.00797)
	2016	0.00953 (0.00397-0.02247)
	2017	0.00151 (0.00062-0.00375)
	2018	0.00111 (0.00044-0.0028)
	2019	0.00397 (0.00173-0.00888)
**AS**	2010	0.00809 (0.00344-0.01844)
	2011	0.00817 (0.00354-0.01825)
	2012	0.00831 (0.00353-0.0189)
	2013	0.00853 (0.00366-0.01959)
	2014	0.00876 (0.00365-0.02018)
	2015	0.00914 (0.00385-0.0211)
	2016	0.01048 (0.00463-0.02318)
	2017	0.08179 (0.04434-0.14784)
	2018	0.04357 (0.02115-0.08797)
	2019	0.00789 (0.00336-0.01786)
**VI**	2010	7e-04 (0.00014-0.00307)
	2011	0.00071 (0.00014-0.00306)
	2012	0.06289 (0.02814-0.12663)
	2013	0.04216 (0.01612-0.10975)
	2014	0.00594 (0.00193-0.01754)
	2015	0.00167 (0.00044-0.00591)
	2016	0.0044 (0.00141-0.01359)
	2017	0.00102 (0.00023-0.00408)
	2018	0.00071 (0.00014-0.00309)
	2019	0.00174 (0.00047-0.00617)
**HI**	2010	1e-05 (0-0.00028)
	2011	0.00246 (0.00017-0.01175)
	2012	1e-05 (0-0.00017)
	2013	1e-05 (0-0.00018)
	2014	1e-05 (0-0.00022)
	2015	0.01685 (0.0052-0.05534)
	2016	0.00113 (0.00036-0.00371)
	2017	0 (0-6e-05)
	2018	0 (0-6e-05)
	2019	0 (0-5e-05)

Table B. Force of infection estimates in Puerto Rico, American Samoa, US Virgin Islands, Hawaii, Florida and Guam from 2010 to 2019. Median and 95 % Bayesian credible intervals. *(continued)*

Location	Year	Force of Infection (95% CrI)
**FL**	2010	9e-05 (2e-05-0.00046)
	2011	1e-05 (0-6e-05)
	2012	0 (0-1e-05)
	2013	4e-05 (1e-05-0.00019)
	2014	1e-05 (0-6e-05)
	2015	0 (0-1e-05)
	2016	1e-05 (0-4e-05)
	2017	0 (0-0)
	2018	0 (0-1e-05)
	2019	3e-05 (1e-05-0.00014)
**GU**	2010	2e-05 (0-0.00069)
	2011	2e-05 (0-0.00071)
	2012	2e-05 (0-0.00072)
	2013	2e-05 (0-0.00076)
	2014	3e-05 (0-0.00119)
	2015	3e-05 (0-0.0025)
	2016	5e-05 (0-0.00448)
	2017	8e-05 (0-0.00589)
	2018	0.00018 (0-0.00711)
	2019	0.00988 (0.00185-0.05343)

Table C. Long-term average of force of infection estimates in Puerto Rico, American Samoa, US Virgin Islands, Hawaii, Florida and Guam for the 1929–2009 and 2010–2019 time periods. The 2010–2019 period corresponds to the period with data.

location	1929-2009	2010-2019
**PR**	0.0111115	0.0102152
**AS**	0.0142019	0.0129584
**VI**	0.0050122	0.0032798
**HI**	0.0000094	0.0000337
**FL**	0.0000119	0.0000058
**GU**	0.0000300	0.0000617

References

Thomas Wutzler. *logitnorm: Functions for the Logitnormal Distribution*, 2021. URL https://CRAN.R-project.org/package=logitnorm.
